# Using reactive links to propagate changes across engineering models

**DOI:** 10.1007/s10270-024-01186-w

**Published:** 2024-06-10

**Authors:** Cosmina-Cristina Raţiu, Wesley K. G. Assunção, Edvin Herac, Rainer Haas, Christophe Lauwerys, Alexander Egyed

**Affiliations:** 1 Institute for Software Systems Engineering, Johannes Kepler University Linz, Linz, Austria; 2 Pro2Future GmbH, Altenberger Strasse 69, 4040 Linz, Austria; 3 Department of Computer Science, North Carolina State University, Raleigh, NC USA; 4 Institute for Software Systems Engineering, Johannes Kepler University Linz, Linz, Austria; 5 Linz Center of Mechatronics GmbH, Linz, Austria; 6 Corelab MotionS, Flanders Make, Ghent, Belgium; 7 Institute for Software Systems Engineering, Johannes Kepler University Linz, Linz, Austria

**Keywords:** Change propagation, Model-driven engineering, Heterogeneous models, Collaboration, Multi-domain traceability

## Abstract

Collaborative model-driven development is a *de facto* practice to create software-intensive systems in several domains (e.g., aerospace, automotive, and robotics). However, when multiple engineers work concurrently, keeping all model artifacts synchronized and consistent is difficult. This is even harder when the engineering process relies on a myriad of tools and domains (e.g., mechanic, electronic, and software). Existing work tries to solve this issue from different perspectives, such as using trace links between different artifacts or computing change propagation paths. However, these solutions mainly provide additional information to engineers, still requiring manual work for propagating changes. Yet, most modeling tools are limited regarding the traceability between different domains, while also lacking the efficiency and granularity required during the development of software-intensive systems. Motivated by these limitations, in this work, we present a solution based on what we call “reactive links”, which are highly granular trace links that propagate change between property values across models in different domains, managed in different tools. Differently from traditional “passive links”, reactive links automatically propagate changes when engineers modify models, assuring the synchronization and consistency of the artifacts. The feasibility, performance, and flexibility of our solution were evaluated in three practical scenarios, from two partner organizations. Our solution is able to resolve all cases in which change propagation among models were required. We observed a great improvement of efficiency when compared to the same propagation if done manually. The contribution of this work is to enhance the engineering of software-intensive systems by reducing the burden of manually keeping models synchronized and avoiding inconsistencies that potentially can originate from collaborative engineering in a variety of tool from different domains.

## Introduction

A fundamental part of engineering software-intensive systems is the set of design decisions that are made based on specific requirements and their realization through various implementation artifacts such as models, prototypes, and source code [78]. However, these engineering artifacts are dynamic and undergo constant refinement, alteration, and updates [24, 34, 54, 74]. As the development progresses, engineers gain a deeper understanding of the project, select various off-the-shelf components, and develop progressively complex prototypes for client review [37, 110]. With each step, the constraints and dependencies between the artifacts, including model elements, are revised, and consistency rules are established [22, 101]. Throughout the entire engineering process, it is crucial for the artifacts to maintain consistency with one another and adhere to the constraints set for the requirements and other related implementation artifacts [22, 101]. Achieving this is not an easy task in general, and it is even more challenging in the context of collaborative, multi-domain, and model-driven engineering [16, 19, 69, 83].

For developing software-intensive systems, regardless of their size, it is common for multiple teams to collaborate [40, 59, 97]. Each team focuses on a specific aspect of the system and uses separate tools to ensure domain-specific properties are met. As a result, different but related artifacts are created [72]. Despite their differences, all artifacts represent the same system and must be synchronized. If changes to the specifications require new property values in one tool, these changes must be propagated throughout all affected dependencies [84]. Furthermore, if new dependencies are affected, they must also be updated accordingly.

The issue of linking artifacts has been examined in the literature from various angles, and different solutions have been proposed to aid collaboration among engineers. Trace links have been created between related artifacts using various methods [7, 25, 66, 85, 96], which can help manage relationships and constraints between models. However, these links are “passive” and do not automatically propagate changes. Consistency rules can highlight inconsistent data [2, 4, 108], but they may not provide the necessary granularity for all use cases, even when paired with traceability. Change propagation algorithms can determine the ideal propagation paths or predict the impact of a change [3, 46, 77, 101, 109], but manual propagation is still required, and these algorithms can slow down the development process due to their high computation times. Only a few solutions can provide automatic change propagation across linked artifacts [29, 32, 36, 43, 70, 87], but they require the use of a limited set of tools and often cannot handle multi-tool and multi-domain systems. These limitations compromise the engineering of software-intensive systems by hiding dependencies or potential inconsistencies, ultimately affecting the quality of the final products.

Our work aims to overcome the limitations discussed above by proposing a solution that integrates traceability [23], consistency checking [39], and change propagation [48], while also considering a finer granularity of engineering artifacts. Our solution uses “*reactive links*” to automatically propagate fine-grained changes, specifically at the level of model properties, across multi-domain artifacts of software-intensive systems. These links establish connections between related properties in different artifacts. Whenever a change in any artifact affects a linked property, our solution immediately applies its impact on all related artifacts, as configured by the users. These reactive links prevent unnoticed inconsistencies that could persist in the system until they lead to severe faults.

In this paper, we investigate three research questions. Firstly, we look into whether our proposed solution can cover all the use cases present in our evaluation scenarios. Then, we investigate whether the reactive links can be flexibly applied in diverse engineering projects. Finally, we discuss the performance of the reactive links in terms of memory and run time. Our solution was evaluated against these three research questions through three engineering scenarios that encompass various domains, tools, and change propagation cases.^1^ The results demonstrate the feasibility and performance of the reactive links in these scenarios, including a notable enhancement in maintaining constraints and consistency among artifacts. We identified potential areas for expanding the links to improve the collaborative model-driven engineering process significantly. Furthermore, we also recognized that there is room for future improvements, and we compiled a list of potential ways to enhance and customize the solution for different use cases.

Our solution’s primary contribution is its ability to substantially minimize the overhead needed to uphold the dependencies and consistency between various models in collaborative engineering. Our solution can assist engineers toward the following benefits: (i) reducing the probability of errors during the modeling process, such as product validation, by identifying any dependency violation among model artifacts and (ii) reducing the risk of models using obsolete or inconsistent data by instantaneously propagating changes as soon as they are detected in any of the artifacts.

This paper is an extension of our previous work [84], specifically, we: (i) consider new link operators to deal with collections and textual values in the properties that need to be propagated; (ii) recommend a process for creating and maintaining the reactive links; (iii) address the problem of circular links and floating point precision; (iv) add a new case study from an industry partner [67]; (v) provide more details of the solution and the implementation used in the evaluation; and (vi) extend the results to evaluate the flexibility and memory impact of our approach.

This paper is structured into sections. First, in Sect. 2, an example of the risks involved in synchronizing related artifacts is introduced. Next, in Sect. 3, a proposed solution is described to address these risks. In Sect. 4, the study design and research questions are presented, along with an explanation of how the proposed solution is evaluated using two engineering scenarios. Then, in Sects. 5 and 6, the evaluation results and potential threats to the validity of the study are discussed, respectively. Additionally, Sect. 7 provides an overview of related work in the literature. Finally, in Sect. 8, the paper concludes with a final assessment of the suitability of the proposed solution for the identified problem.

**Fig. 1:**
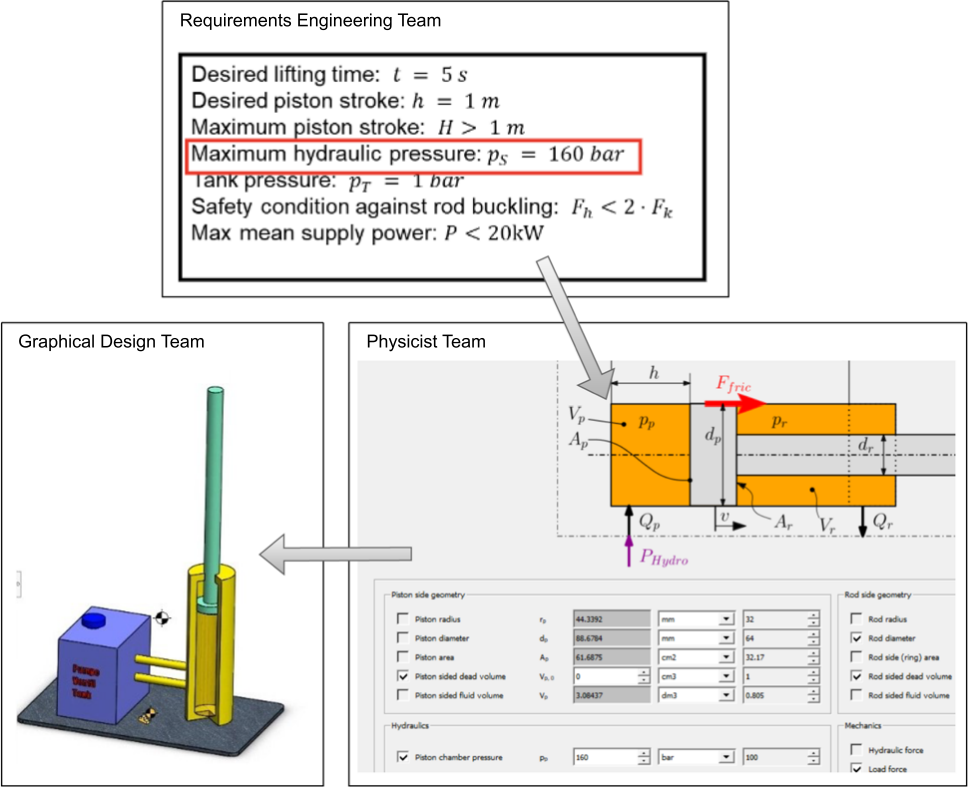
Representation of the teams, tools, and model elements involved in the motivational example

## Motivational example

In this section, we present an illustrative example that demonstrates the risks involved in synchronizing related artifacts. This example is extracted from one of the engineering scenarios of our subject systems (described in detail in Sect. 4.2).

Let us consider the development of a hydraulic actuator, illustrated in Fig. 1, which involves three teams working collaboratively. The first team, depicted on the upmost part of the figure, comprises *requirement engineers* who use a requirements tool. The second team consists of *physicists* who use a complex calculator to determine the parameter values that best describe the system. This team is represented on the right-hand side of the figure through a capture from their calculator worksheet. The third team, on the left-hand side in the figure, comprises *graphical designers* who create prototypes of system iterations using a graphical modeling tool. This example is illustrated in Fig. 1.

To provide more context for the example, let us take a closer look at the hydraulic actuator being developed. The actuator consists of a cylinder with a piston that is connected to a pump. For the purposes of this example, we focus solely on the cylinder. One of the requirements for this cylinder, marked in Fig. 1 with a red frame, specifies that the pressure within it should not exceed the atmospheric pressure, which is a maximum of *160 bar*. This threshold is critical for ensuring the safe and accurate operation of the piston. If this number is significantly higher than the maximum pressure within the cylinder, the client has to needlessly spend much more on the components of the system. On the other hand, if the pressure within the cylinder ever exceeds this specification, the hydraulic actuator is likely to become unsafe and unusable.

The team of physicists responsible for designing the hydraulic actuator uses a dedicated calculator worksheet for the cylinder, which takes pressure as input and calculates other significant data such as the diameter of the piston cylinder. The goal of the team is to find the minimum cylinder diameter that ensures the safe operation of the actuator while taking into account the threshold value for pressure. The resulting diameter is then communicated to the graphical modeling team, who use it to update their design. The updated design includes threshold values that are communicated to the client when selecting off-the-shelf parts.

### Problem statement

Ideally, the three models upon which the development of the hydraulic actuator is based would be fully synchronized. The dependencies between these models are represented in Fig. 1 with gray arrows. However, this is not often the case in practice, particularly when teams with varying levels of insight collaborate on different models. Human error is not uncommon, and if each team is solely responsible for one tool and one model, violations of constraints and inconsistencies may go unnoticed.

As an example, let us assume that a mistake occurs when the maximum required piston pressure is entered, resulting in a value of only *60 bar* in the calculator worksheet. This value is then used to determine the piston diameter, resulting in a system that is actually unsafe. Despite the requirement constraint of a maximum of *160 bar*, engineers incorporate this value into the graphical design without realizing the mistake. However, neither the requirements engineering team nor the graphical modeling team has the insight or responsibility to verify the correctness of the values obtained from the computation. This incorrect value can remain undetected in the system until the quality assurance team notices the issue late in the development process and reports it. Correcting the mistake requires the collaboration of all the teams involved and additional reviews, which lengthens the process and increases the costs and effort required. If left undetected, the mistake could result in the client acquiring improper and unsafe components based on incorrect information.

### The limitations of the existing solutions

This section provides a brief overview of the existing solutions proposed in the literature to address the problem, which are discussed in detail in Sect. 7. However, it is important to note that these existing solutions have their own limitations and do not fully address the problem at hand. While each solution may solve a part of the problem, none of them provides a complete solution, and each has its own drawbacks.

#### Trace links

Two trace links can be utilized to establish a connection between (i) the pressure requirement and the computation and (ii) the computation result and the cylinder diameter in the graphical design. However, there are two major drawbacks to this approach. Firstly, the two relationships represented here have different roles. In the case of (i), the aim is to maintain consistency with a *threshold*, while in the case of (ii), the properties’ values are expected to be *equal*. The engineers can either create two different trace matrices only for these cases, or choose general trace types that do not provide all the necessary information. Secondly, the traces are merely passive links that cannot propagate information or verify constraints, they only indicate the relationships between artifacts. Ultimately, it is still the engineers’ responsibility to ensure the constraint fulfillment.

#### Consistency rules

Another option for engineers is to establish consistency rules. However, this approach requires a way to connect artifacts across tool boundaries. If the system can be reduced to one metamodel, this is not a problem. But when connecting artifacts across models, consistency checking can be combined with traceability to maintain most of the downsides discussed earlier, particularly the lack of granularity.

Trace links are defined at the artifact level, such as connecting requirements to the calculator artifacts that use them. However, since there is only one requirement that specifies the maximum piston pressure, setting a consistency rule using traces can result in latency and redundancy. Every trace is checked for a parameter that is only specified once, potentially resulting in false inconsistencies for requirements that do not specify a maximum piston pressure. Furthermore, the rules must be carefully defined to avoid false results. Marking a requirement as inconsistent just because it does not, and should not, specify a piston pressure can create more problems than it solves.

#### Change propagation

Assuming that engineers have a change propagation algorithm to handle the system under development, this is still a challenging task, especially when dealing with multi-domain data. There are two key issues associated with these algorithms. Firstly, change propagation algorithms can be computationally expensive and time-consuming. They typically require an overview of the system in the form of a matrix with all the dependencies between artifacts, which must be parsed to simulate the propagation. This process can be particularly taxing for even small systems, such as the hydraulic actuator, which involve a significant number of artifacts. Secondly, change propagation is not always automated. While propagation algorithms can suggest ways to handle constraints and resolve inconsistencies, engineers must still manually apply the necessary changes. This manual intervention prolongs the overall time required to return the system to a consistent state and does not fully address the risk of human error.

#### Automatic knowledge propagation

Automatically propagating links are an appealing solution in theory for resolving inconsistencies between computation results and graphical designs. However, in practice, the solutions proposed in this area are often domain-specific. For instance, many of the available solutions are limited to systems described entirely using Unified Modeling Language (UML), which is not the case for the problem at hand. While some solutions not dependent on UML exist, they are typically tailored to narrow domains and cannot handle the combination of artifacts present in our scenario, which includes requirements, computations, and graphical elements.

One limitation of the existing solutions for propagation links is their inability to handle constraint violations. Although they may ensure that values remain consistent, they cannot address constraints such as the maximum piston pressure requirement in our example. This creates a gap in the system’s ability to ensure that all constraints are met and increases the risk of unsafe or improper components being produced. A more comprehensive approach is needed to address both consistency and constraint violations in the system.

## Proposed solution

Our solution aims to mitigate the primary shortcomings of the existing solutions discussed in the section above, while leveraging and improving on their respective advantages.

### Working assumptions

Before our solution can be considered feasible, the environment and use case must fulfill a set of conditions, which we assume to be true.

Firstly, one of the main features of the reactive links is specifically their fine granularity. They can connect two artifact property values and check their consistency with each other or propagate changes from one value to the other. This feature makes reactive links not feasible for coarser traceability, change propagation, and consistency checking use cases. Thus, we assume that the purpose of the use of the reactive links is to create and synchronize fine-grained values stored in specific properties of the artifacts.

We also assume that the models to be linked can be stored in a common environment with a uniform and extensible artifact representation. This environment can be any technology that allows multiple tools and models to be stored together and extended with additional information. These aspects are discussed in more detail in Sects. 3.2 and 4.3. Besides storing the data, we assume that this common environment offers a mechanism of communication with the original tools in which the models were developed. This mechanism serves to ensure that the models in a tool are consistent and synchronized with the information in the common environment. The specific representation of the data (e.g., UML format) and the tool-side view of it are not particularly important as long as the common environment representation supports the addition of reactive links.

### Artifacts representation

**Fig. 2:**
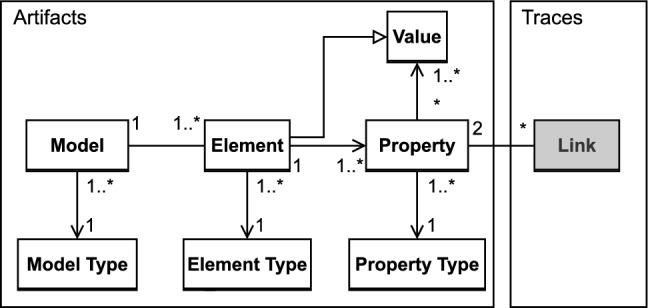
Representation of multi-domain models and links

As stated above, the majority of knowledge propagation solutions necessitate a UML representation. However, our solution is tailored to function in a scenario where multiple domains and tools are employed, as described in the motivational example. To achieve this, we established a generic representation, presented in Fig. 2, which can handle various types of models and artifacts.

Figure 2 presents a metamodel defined to represent the artifacts in a generic way. On the left side of the figure, the metamodel describes the elements to represent the artifacts/models. On the right side, the links added by our solution are shown. This metamodel is based on a model type that contains model elements. Each element is assigned a type and has properties, which in turn have a type and a set of possible values. Elements are a subclass of values, as a model element can also be a value. With this approach, we aim to provide a flexible and adaptable representation of artifacts that can be used across multiple domains and tools, regardless of the type of model being used.

**Fig. 3:**
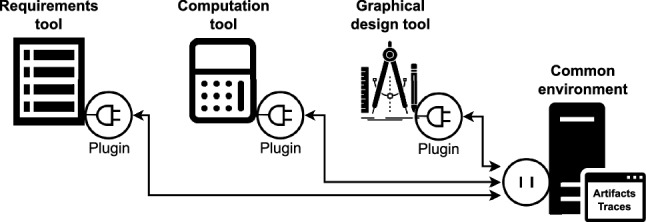
Common environment with multi-tools and plugins

To allow the specification of the links, the artifacts to be linked need not only to be represented according to this metamodel, but also to be stored in a *common environment*. As illustrated in Fig. 3, this common environment provides an integration between the different tools used in the toolset. This is required to allow the artifacts created and managed by different tools to interact with each other, while also providing an overview representation of the cross-domain modeling information. In the absence of such an environment, the artifacts are only visible within their original tools, which is a problem already discussed in the example (Sect. 2). In this case, our solution cannot obtain or provide any knowledge to related artifacts in other sections of the model.

To bridge the gap between the individual tools and the common environment, each tool has to be equipped with a plugin or integration component that would enable it to communicate with the common environment. This component must fulfill three tasks:

1. Translating the artifacts from their in-tool representation to our metamodel (Fig. 2), so that the artifact data and any subsequent changes to it are visible in the common environment.
2. Translating any changes of their artifacts from the new metamodel back into the tool metamodel, so that tool users can access any changes to the data stored in the common environment.
3. Continuously observing and applying any changes that occur to the data, so that the two representations of the data are synchronized.

Details of the implementation for the *common environment* and the tools equipped with plugins are presented in Sect. 4.3.

### Linking Granularity

As discussed in Sect. 2, trace links establish connections at the model level. However, the target information of these links is usually stored at the level of property values. This means that changes to other properties that are not relevant to the links may still trigger propagation or consistency checking, even when they have no actual impact on the linked details.

Unlike existing trace links, which operate at a high level of abstraction and may trigger unnecessary propagation or consistency checks, our solution enables engineers to link specific properties that have a correspondence. For example, in our illustrative example (Sect. 2), rather than linking the entire requirement to the computation, our solution allows engineers to link only the property that stores the constraint value (160 bar) to the corresponding property in the computation. This granular approach ensures that only changes to relevant properties trigger link execution, while other changes do not impact the links, therefore not slowing down the system with unnecessary link executions. On the right side of Fig. 2, we show that a link is associated with two properties, which enable the traceability with finer granularity. More implementation details are provided in Sect. 4.

### Propagation operators

To ensure that the linked properties are updated appropriately, it is necessary to define the corresponding operation that will be triggered upon a relevant change. However, existing solutions suffer from a significant limitation in that they are designed to handle only one specific operation. This can range from simply updating values directly to flagging constraint violations, which must then be manually resolved by engineers at a later stage. This greatly limits the application of such solutions for different practical scenarios, as discussed in our motivational example.

Our solution combines both constraint checking and change propagation into a single mechanism. When setting a link, the user selects the operator to be applied after a property change. An *operator* determines which action is automatically performed on the linked properties. Some operators can also check whether a requirement constraint or consistency rule is violated. Table 1 presents a selection of the link operators available in our solution, specifically for numerical property values.

**Table 1:** Operator to define the link actions

Operator	Name	Description
$=$	Assignment	Propagates the change to the linked property value
${=}{=}$	Equals	Checks the equality of the linked values
${!}{=}$	Not equals	Checks the inequality of the linked values
<	Less than	Checks that the value is less than its counterpart
${<}{=}$	Less than or Equal	Checks that the value is less or equal to its counterpart
>	Greater than	Checks that the value is greater than its counterpart
${>}{=}$	Greater than or Equal	Checks that the value is greater or equal to its counterpart

**Table 2:** Operator for properties of type Set and String

Operator	Description
includes	Checks that one set property value contains all the elements of another set property value
contains	Checks that the set property value contains one specific property value
startWith	Checks that a string property value begins with the characters of another string property value
endsWith	Checks that a string property value ends with the characters of another string property value
subString	Checks that a string property value contains another string property value

Of the operators presented, the assignment ($=$), equals ($==$), and not equals ($!=$) can be applied to any property type, ranging from numbers to reference properties for which the value is another model element. Additionally, our solution can be extended with other specialized operators depending on the property types encountered in the models to which it is applied. Each data type, such as a set, or a number, has several specific characteristics to be considered when checking the consistency of dependent properties. For example, one number can be greater than another, checked with a > operator, but the same operator is meaningless for sets. The opposite is true for the “includes” operator, which applies to collection-typed properties, but not to numerical values. In order to increase the flexibility of our solution, we have extended the operators list with a selection of set-based and string-based operators, such as the “includes” operator, as seen in Table 2. Depending on the prevalent data types and dependencies in the target system, our solution can be extended with type-specific or custom operators, the semantics of which focus on these specific cases.

### Propagation direction

While designing the link operations, we also realized that, in many cases, the execution of the link depends on which end of the link was changed. For instance, considering the hydraulic actuator discussed in Sect. 2, engineers can set a link connecting the piston cylinder diameter in the computation and the graphical model. They expect that the value in the graphical design always corresponds to the computation, so they choose an assignment operator ($=$) while creating the link. However, the piston diameter in the computation is a result and can only be changed if the input changes.

The expected reaction to a change can further differ depending on the artifacts linked. In the case of linking computational output and the graphical model parameters, the users may want to leave room for experimentation. If the computation input changes, the new result will be propagated immediately. But the graphical model may be used in the communication with the client, during which the client may request different ad hoc experiments that temporarily invalidate the dependency between the two properties. In this case, the graphical designers may want the changes to only be visible on their side of the model, since new input values will later override them as soon as the feedback from the client interaction is applied to the model.

From a different perspective, going back to the initial example, the model may have certain constants or stead-fast parameters specified in the requirements, which are then used during the computation. If any of these parameters change in the requirements, the change has to be mirrored in the computational model. However, if one of the physicists working on the computation accidentally changes one of these values, they would not expect the requirement specification to be changed accordingly. Quite the opposite, they would expect this discrepancy to be immediately flagged as an inconsistency in the overall model.

Finally, for the sake of completeness, the model will most likely contain instances where the engineers will expect bidirectional propagation to occur. This can happen in two situations: (i) when applying a constraint checking link, for example, using a “greater than” operator, or (ii) when the two corresponding values are both editable input values in two different tools or artifacts. This second case can occur in tools that are not well integrated, such as when the same value is required in two different computations that the calculator tool cannot link. For example, the dimensions of the piston are required both in the computations regarding the cylinder properties, and in the computations that determine the safety parameters of the piston rod. Alternatively, one value could be required as an input in two different tools and expected to be synchronized regardless of the source of the change. For example, the length of the piston is required both in the computations and in the graphical model, but the value should always be equal between the two. Unlike the diameter of the cylinder, the piston length is not open to experimentation in client communications.

The links should, therefore, hold the knowledge about the *direction* of propagation. This means that the link will react depending on the origin of the change. Table 3 presents the three propagation directions supported by our solution, which fully cover the various scenarios described above. The engineers must decide the trigger direction for each link during the link creation process. However, if the editing of existing links is enabled, this direction can be changed at any point during the development process.

**Table 3:** Direction of the link operations

Name	Description
Bidirectional	The links are triggered and evaluated identically for changes in both properties
Unidirectional	The changes are propagated in one direction of the link, and ignored in the opposite direction
Asymmetrical	The link evaluation is different depending on the changed property value

### Additional propagation transformations

Although direct propagation or constraint checking can satisfy many use cases, some require additional transformations. For instance, the hydraulic actuator requirements impose a constraint for safety against rod buckling. The constraint requires that the hydraulic force be less than twice the buckling force, both of which are computation results in different calculator worksheets. Implementing this constraint with a simple link would necessitate an intermediate property value that computes the double of the buckling force. Our linking solution allows engineers to specify *simple transformations* to be applied during link execution. In this scenario, engineers can add the “2*” operation during link setup, and the operation will be performed after each relevant change.

These simple transformations can vary from small computational changes, such as the example above, to type changes and aggregation operations for collection-type properties. For example, such a transformation could be used to parse a textual requirement description for the numerical specification of a constant that is required in the computation. Or alternatively, as will be captured later in the evaluation (Sect. 5), a computation result from the calculator can be automatically converted to a string and inserted into the Java-based source code of a component.

### Propagation triggers

**Table 4:** Triggers to define when the links are executed

Name	Description
Automatic	Whenever a property value changes, all the links with these triggers are automatically executed
Manual	The user decides when to manually trigger the execution of the links

Another design decision of our solution is when exactly the link actions are triggered. Table 4 presents the two kinds of *triggers* supported in our solution. The *manual* propagation is the least intrusive, but requires more user interaction and care. While it allows the engineers to maintain closer control of the change propagation, every link has to be manually activated. As a result, impactful changes could still be overlooked. Even worse, some value propagations might be incomplete, which could happen if the propagation of a change results in a subsequent change. For example, a change in a requirement-specified constant requires the engineers to manually trigger a propagation link to propagate the new value to the computation. But this input change could result in a new computation result that, if involved in one or more links, would require the engineer to manually trigger those links as well.

The option of *automatic* propagation requires no user intervention after its creation and allows for chain propagation. In chain propagation, one link evaluation triggers another to ensure that the full impact of a change is propagated throughout the system without any additional user input. This can prevent cases in which the change propagation is incomplete, but it may reduce the control engineers have on the propagation. The result is the possibility that intermediary or experimental values are propagated throughout the system, when the engineers only intended to test them locally.

There are advantages and disadvantages to both options, which should be considered and balanced with respect to the engineers’ goals, the types of artifacts involved, the criticality of the requirement constraints or consistency rules, and the implementation details.

### Type-specific challenges: floating point precision

The reactive links can, as previously noted, propagate changes and check for simple inconsistencies between a wide variety of data types. However, each data type may have specific features and challenges to be considered for a proper implementation of the linking system. One challenge is the floating point precision in real number computations.

Computations containing varying floating point precision levels are very common in practice [64, 89]. In the context of our motivational example, the precision levels differ from one tool to the next. In bridging the gap between different tools for effective cross-tool collaboration, precision levels add another layer of complexity. If the reactive links do not consider this problem, this may lead to rounding errors. These can cause issues from propagating inconsistent data and generating incorrect computation results, to inadvertently creating endless propagation loops if each tool performs a slightly different rounding on the same property value. In order to prevent this situation, the reactive links can be paired with floating point precision identification and tuning solutions. These may be different forms of static analysis [42] to identify possible precision-induced faults before they affect the system. Alternatively, a wide variety of solutions have been proposed over the years to algorithmically [6, 21, 26, 114] or automatically [63, 64, 89, 90] compute the lowest precision level necessary for an accurate and fast result in mixed-precision computations.

The pitfalls that may result from ignoring this aspect may be preventable by using unidirectional links, carefully avoiding circular propagation paths, and additional checks to ensure the propagated values are correct. However, these conditions are hardly achievable in a real-world, incrementally evolving system. Hence, we recommend that an approach to handle heterogeneous floating point precision is taken into consideration if reactive links are to be used in practice for numerical values. The specific choice as to which of the above approaches is best suited to be used in combination with the links will depend greatly on the other implementation aspects considered, as well as the specific characteristics of the context in which they will be used. For our prototype, we have decided to disregard this issue, as we will further detail in Sect. 4.3.4.

### Addressing link circularity

Independently of the data types linked, one potential issue are circular dependencies. These occur when a selection of property values within the model are all interconnected in a cyclical network. Collaborative systems developed by highly heterogeneous teams and tools may feature large numbers of links between all the different artifacts involved. Additionally, new links are created throughout the development process, as new artifacts and properties are modeled. On top of that, in the cases when a property value appears in three or more artifacts, the changes to this property value have to be propagated in all directions, regardless of where the change originates. Thus, preventing cycles in the link network becomes a complex task.

Circular dependencies predominantly affect automatically triggered links. By default, an automatic propagation across one link will trigger all the links connected to the property value that was changed as a result. Therefore, for automatically triggered links, a circular dependency is likely to cause an infinite propagation cycle. In this case, each link triggers the next in the cyclical network. As a result, the propagation gets stuck in going around this loop. Moreover, in the absence of a solution to this circular propagation issue, this infinite propagation cycle can even occur in bidirectional propagation links, i.e., bidirectional links with the “assignment” ($=$) operator, where the same value could end up being propagated back and forth across one link forever.

A simple solution specifically to this last problem is to prevent a propagation event when the value of the target property value would not change. For example, if the propagation link wants to assign the value “1” to a property value that is already “1”, the propagation would make no effective difference, so it can be skipped. Therefore, both in a bidirectional propagation link and in a cyclical link network, the propagation would stop when a full round of propagation is completed.

There are some cases that might still fail despite additional checks. This could happen when we use simple transformations in the links, which change the value propagated so that a full round is never identified. One example of such a situation is the previously mentioned case concerning varying floating point precision levels between tools, in which rounding errors may cause endless propagation.

The linking service can also prevent circular infinite propagation cycles by detecting and managing circular links. In the landscape of reactive links, we can consider the artifact properties to be vertices in a graph, with the links acting as directed edges. Hence, for the purpose of identifying circular links that may lead to propagation loops, we can consider the network of links to be a directed graph.

Providing a visualization of the linking network can also support engineers in avoiding the creation of cyclical link networks. Even in cases where one person is solely responsible for creating and maintaining the reactive links, a large network of links can quickly become overwhelming. Due to the similarity to directed graphs, different visualization techniques [12, 103] and tools [1, 9] can be adapted to represent a link network overview. Additional interactivity [33, 106] with this overview provides even more support to the engineers. However, manually checking and examining the spread of links in the system for each new link created may still be time-consuming and overwhelming, depending on the size and distribution of the network. Several surveys based on graph visualization offer some insights into supporting engineers through this task [10, 44, 52].

Another alternative is to enhance the linking system with logic that detects and prevents circular paths in the link network without needing additional human input. As the linking system already stores all the links, different graph-based algorithms can be used to examine these links for cycles. Such algorithms are based on the concept of strongly connected components, namely subgraphs in which each vertex can be accessed from each other vertex [104]. The solution is to identify all the strongly connected components in a graph and recursively replace them with a placeholder vertex, resulting in an acyclic graph [47]. The accurate and efficient identification of cycles and strongly connected components has been an active area of research for decades, with multiple algorithms being proposed both for static graphs, where all the edges are known, and for dynamic graphs, where new edges are added incrementally. For static graphs, the most common solutions use a depth-first search [104], which aims to parse the same vertex twice, hence finding a cycle. Alternatively, repeatedly deleting each vertex that does not have a predecessor results in a graph formed solely of strongly connected components [61]. When the edges are added dynamically, these approaches can be applied repeatedly after each new edge is added. However, the field of incremental cycle detection and strongly connected component management contains extensive variations that improve the efficiency of these algorithms. Most of these approaches enhance the algorithms with topologically sorted vertices [11, 13, 45], two-way searches [45, 81], or other methods to reduce the search space necessary to identify possible new cycles [14, 81].

Different approaches are proposed to potentially reduce cycles [49, 98, 105], which can also be employed and adapted to manage existing cycles and prevent, where possible, endless propagation loops. Namely, reducible cycles [49] can be partitioned into two acyclic subgraphs. One is an acyclic subgraph formed by the edges and vertices accessible from the source vertex, which, in our case, could be the property value that was changed to trigger the propagation. The other is formed by the edges with vertices in topological order. While not all cycles are reducible [49, 98], in some applications not all cycles can, or should, be prevented. For example, strongly connected components in the link network might be desired in certain complex multi-tool development environments where the changes have to be propagated regardless of their artifact of origin. In these cases, reducible loops can be used to stop potential endless propagation loops.

Similarly to the issue of floating point precision, the most appropriate mechanism to prevent or manage cycles in link networks will heavily depend on the context in which the links are used. While some users will want to prevent cycles altogether, others may want to allow them but be able to manage or visualize them. Equally, the performance cost of the cycle management must also be considered. In our prototype, we have chosen to implement the algorithm proposed by Tarjan et al. [104], in which we are performing a depth-first search in order to identify any potential cycles before each link is added to the network. We present more details on this implementation choice in Sect. 4.3.4.

### Linking process

Using reactive links helps the engineers automate the management of dependencies and change propagation within their complex development systems. While this reduces the overhead of constantly checking the consistency of the model, the benefits of the links have to be balanced with the drawbacks of creating, managing, and evaluating the network of links. The creation of the links is akin to identifying dependencies in the system, and therefore, the existing process applied can easily be expanded to include this step. However, as each team and company has specific process definitions according to their needs, priorities, and the type of products they develop, the exact linking process will also vary accordingly. In the following, we are presenting our recommendation for a possible linking process.

We assume that the development teams already follow a process that includes an early analysis and identification of the dependencies between models. This is often the case in safety-critical processes, as hidden or lost dependencies may quickly become significant safety risks. This process is usually triggered by a set of new requirements received from the client. For exemplification, we use the scenario introduced in Sect. 2 and consider the requirements shown in Fig. 1 to be the new set of requirements coming from the client. The requirements engineering team elicits and specifies these new requirements. Then, the new design is modeled, followed by a preliminary requirements analysis, at which point dependencies and traceability links can be identified. We propose that this is the step where most of the links are created as well.

The dependencies identification step is often the hardest step in the process, while also playing a crucial role in traceability identification and further safety and consistency checks. In smaller projects, the engineers can conceivably analyze the artifacts and check for dependencies manually. However, this task can easily become not only time-consuming and tedious, but also overwhelming and error-prone. According to various studies, such as Wang et al. [111], the pairwise comparison of all the requirements in a project can take around 12 h. In the case of model-driven engineers, this estimation would also need to be extended to include the artifacts that are part of the models, and for fine-granular dependencies, even the collection of properties each of these artifacts has.

To mitigate this burden, a variety of solutions offer automatic support for dependency identification. Most of these solutions are based on content analysis [65, 79, 91, 111], where the text associated with the artifacts is automatically processed to find common concepts that may correspond to dependencies. Other solutions, such as Savić et al. [92], parse the concrete syntax tree enriched with additional information. Likewise, multiple solutions use different annotations [99, 113] or the results from tracking the engineer activity [62] to increase the accuracy of the dependency prediction. It must be noted that most of the available solutions are developed with a focus on requirements or code dependency identification. While many are applicable in the context of heterogeneous model-driven engineering as well, they may require additional or specialized training data and development. Additionally, for most of these solutions, a dependency is synonymous with a trace link, connecting two artifacts, instead of artifact property values. As a result, the automatic identification of reactive links would either need to be trained to find more fine-granular dependencies or be used in combination with a manual effort from the engineers. Even in the latter case, the use on an automatic dependency identification solution would greatly improve the workload associated with identifying reactive links.

In terms of the process of setting a specific link, after the dependency is identified, translating it into a link is straightforward. The engineers need to define which are the two property values linked, and the behavior of the link is defined by the link properties described in the above sections. In our example, they would select the maximum piston pressure value specified in the requirements and the property describing the same concept in the parameter of the complex calculator. They would connect these two property values through a reactive link and select the appropriate operators. We can assume they would want an asymmetrical link that propagates changes from the requirements into the calculator and detects inconsistencies in the reverse direction. Therefore, they would choose the assignment operator(“=”) for the propagation direction and the equality operator(“==”) for the reverse. Most of the information necessary is already discovered when the dependency is identified. Additional reasoning may be needed depending on the features of the plugins and the source of the property values, for example, whether they are editable. However, this reasoning would also be necessary in the absence of the links and would need to be repeated every time a change affects the dependency.

After the reactive links are created, the development process proceeds as usual. In our working assumption, after the specification of the new requirements and design modeling, the engineering teams begin the development of the new tasks. During this development, new dependencies may be identified. We recommend that these are captured in reactive (or trace, as may be the case) links as soon as possible, to avoid incomplete or incorrect dependencies later on. The development process is usually iterative, with a few new artifacts being created and developed at a time. As a result, we expect that the identification and capturing of new links during the development time is a less daunting task than the initial dependency identification step. Hence, this can be performed manually, or using the same set of solutions as for the previous analysis step.

The link management is even more straightforward in most cases. After the link properties are identified and the link is set, the engineers’ intervention necessary depends only on the propagation trigger selected. For automatic propagation, no further intervention is necessary at all, as the link will automatically trigger every time the dependency is touched by a change. For manual propagation, engineers have to manually trigger the links. However, this operation can be done as part of the commit process, during which engineers acknowledge their changes are complete and should be visible to the other teams working on the same project.

When a link becomes redundant or needs to change its function, the interaction necessary also depends on the trigger selected. If the engineers do not need any more propagation to occur along one dependency with manual triggers, no interaction is needed. The link can be deleted, but it can also be ignored and no longer triggered. For automatic propagation, the engineers have to manually edit or delete the link.

Lastly, the reaction, namely the actual propagation operations through the links, is already optimized as a result of the link design. In automatic triggering, the links are only evaluated when a change directly affects one of the property values connected. As a result, the links will never be triggered unless propagation is really necessary, in cases engineers would otherwise have to manually change the model elements themselves. Additionally, the links represent simple operations, ranging between direct propagation and simple constraint checking. Even the transformations supported are quite simple since we are only connecting two values in the model. Therefore, the actual evaluation of the link is not expected to be significantly resource-consuming.

## Study design

The goal of our study is to evaluate the proposed solution in terms of feasibility, flexibility, and performance. In the following, we present the research questions, three different engineering scenarios, and the implementation aspects of our solution.

### Research question

Based on the goal described above, we have defined three research questions (RQs), as follows:

*RQ1: Feasibility-Does the proposed solution cover all linking cases of our three multi-domain and multi-tool scenarios and keep artifacts synchronized? * We focus on exploring three scenarios in order to evaluate the behavior of our solution when dealing with a wide variety of constraints and consistency rules between different models. We observe the change propagation as part of three scenarios and check the correctness of the result.

*RQ2: Flexibility-To what extent can the proposed solution be applied in diverse engineering projects?* Different engineering project involves different tools and model types that need to be linked. To evaluate the flexibility of our solution, we study the behavior of the reactive links in the context of different property value types. For this, we identify dependencies between different property types that are captured in our engineering scenarios and explore how the links can be applied in each such context.

*RQ3: Performance-How do the reactive links influence the performance of the system in terms of memory usage and run time as compared to manual propagation?* To evaluate performance, we first discuss the engineering effort necessary to set up and maintain the links in comparison with manually propagating changes. After this, we compare the number of artifacts added by the reactive links as compared to the model artifacts contained in each of the engineering scenarios considered. Then, we measure the performance, in terms of execution times, of the links in order to determine whether they provide a significant improvement in comparison with the scenario in which developers manually maintain the cross-domain consistency of the system.

### Subject systems

For evaluating our solution, we have considered three engineering scenarios. The name, number of domains, artifacts, properties, and links for each scenario are presented in Table 5. The scenarios *Hydraulic actuator* and *Robotic arm* have three and five domains, i.e., different numbers of engineering tools, respectively. They were developed by our collaborators working for Linz Center of Mechatronics based on real-world projects they have contributed to in the mechatronics industry. The scenario *Agricultural machines* has two domains that have to be synchronized after each change during the development. This scenario was developed by our collaborators working for Flanders Make, based on an ongoing project with their industry partners. Altogether, the scenarios of our study have seven distinct types of artifacts (i.e., models) managed in different tools: requirements, physical computations, graphical design, coordinates in a spreadsheet, source code, interface definition on GitHub, and UML-like system design.

**Table 5:** Details of the scenarios

Scenario	# Domains	# Artifacts	# Properties	# Links
Hydraulic actuator	3	293	1719	17
Robotic arm	5	981	14836	53
Agricultural machines	2	175	1597	28

**Fig. 4:**
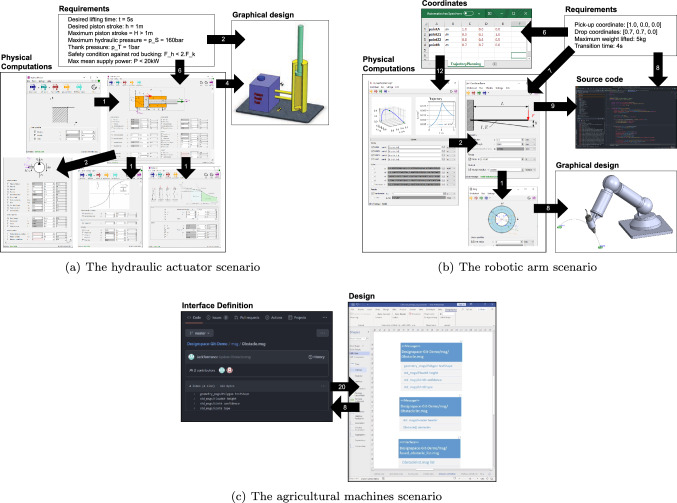
The scenarios, artifacts linked, and the number of links used for the evaluation

The number of links in Table 5, totaling 98, provides us with a diverse range of types, constraints, and rules. This diversity of links enables us to thoroughly evaluate our solution. The requirements for these links were provided by either engineers or clients. Further details regarding the various domains and artifacts can be found in Fig. 4. This figure also shows the number of reactive links that exist between different types of artifacts. In the subsequent section, we provide a detailed explanation of the three scenarios.

#### Hydraulic actuator

This scenario^2^ consists of a piston within a hydraulic cylinder, a pump, and a valve system assuring the connection between the two main components. For developing this project, the engineers model the pump starting with the initial requirements. Next, the teams go through iterations of model refinement. Finally, the engineers check that the building blocks of the system follow the requirement constraints and the safety conditions. There were three teams that collaborate in the engineering process, each with a separate set of responsibilities and expertise, as follows:

- *Requirement engineers* using our requirements tool.^3^ This tool allows these engineers to specify and manage the requirements, in which they can specify quality measurements and constraints. In the context of the common environment (see Sect. 3.2), these additional constraints are stored as custom property values associated with the requirement they are part of.
- *Physicists* use TechCalc,^4^ a computation calculator that has worksheets corresponding to the components of the system. Engineers select the input parameters in the worksheets, and the calculator applies the appropriate formulas to compute the rest of the parameters to characterize the components.
- *Graphical designers* use SolidWorks^5^ to manage a virtual model of the system, visible on the right of Fig. 4 (a) based on the data received from the other two teams.

The actuator development process has four stages:

(i) *initial setup*, the requirements are collected and the preliminary computations and graphical components are set up;
(ii) *threshold computation*, the values of the constraints are used to determine the most extreme component measurements for which the system works safely, as presented in our motivating example;
(iii) *model development*, the teams model the system based on the requirements and the progressive feedback they receive from the client;
(iv) *selection of the off-the-shelf components*, a multi-stage process in which the client selects off-the-shelf components for the project and communicates the characteristics to the teams, then engineers ensure the components will interact correctly.

#### Robotic arm

This project^6^ consists of a factory setup composed of several robotic arms that should pick up an object from the respective receiving bays and transport it to the target locations. The development is based on a basic setup, which is then adapted to the factory requirements provided by the client. For the development of this product, the collaboration is more complex. While the hydraulic actuator consists only of hardware, the robotic arm also requires a software program to control its movement. Therefore, the teams consist of:

- *Requirement engineers* using the same requirements tools as the previous scenario.
- *Mathematicians* translate the real-life setup of the bays and movement trajectory into relative coordinates respective to each robot base, stored in Microsoft Excel.^7^
- *Physicists*, also using TechCalc, with a focus on the movement of the robot and the components necessary for the correct trajectory and functionality to be achieved.
- *Graphical designers* produce visualizations of the arm using SolidWorks.
- A team of *software engineers* who write and maintain the code to control the robotic arm’s movement. The code is written in Java, using the editor IntelliJ,^8^ and provides a basic setup of two different controllers. The most appropriate one is selected through configurations.

For this evaluation, we focused on the steps required for the basic setup to be specialized toward the specific requirements of a client. The models of the system are set up, including the correspondences between the artifacts. The requirements specify the absolute coordinates of the loading bay and the drop point, as well as the robot base location and the requirements for its functioning for each robotic arm. The mathematicians then compute the coordinates from the perspective of the robot, which will influence the computations. The result of the computations specifies the components required for the building of the robot, as well as the deflection of the arm, which is necessary to select the most appropriate controller code for each robot.

#### Agricultural machines

This scenario comes from the agricultural domain, based on the development process of an industry partner of Flanders Make [67]. The agricultural machines built by this company are composed of hardware components (e.g., sensors) and software components (e.g., embedded systems). The communication between these two types of components is assured through message interfaces. During the development process, two collaborating teams develop these separate, but tightly-knit parts of the product. As a result, the message interfaces used for the communication between these two parts must be synchronized and maintained by both teams. The process layout is as follows:

- *Software engineers* use GitHub as a tool to collaboratively develop the software code for the product, as well as maintain the communication interface on the software side. The interfaces here are modeled as Robot Operating System (ROS) protocol [82] files, containing the expected attributes’ type and name.
- *Mechatronics engineers* use Visio-based UML diagrams to model the product from the hardware perspective, including the connections and actions of the sensors involved, and the interface through which the sensor activity is communicated to the software components. These are modeled as UML classes, with each attribute as a separate field.

The industry synchronizes the interfaces using naming conventions. In our evaluation, we extend the two models with two types of links. Firstly, we add links that check that the type and name of each attribute are identical, to highlight any naming convention violation. Then, we add a propagation link for each interface, which automatically adds new required fields from the software interfaces into the hardware models.

This additional scenario allows us to better evaluate the flexibility of the reactive links. On the one hand, unlike the other two scenarios that are heavily focused on numerical property values, the Agricultural Machines feature strings and sets. The reactive links are extended to handle these additional data types and use cases, by simply adding new operators. As such, the links can be easily applied in a completely different setting with very minimal changes required, if any change at all. Additionally, the two tools used in this scenario are completely different from the toolset used in the other two scenarios. The new adapters involved, particularly the GitHub adapter, function differently from the ones we have used in our previous evaluations. As described in Sect. 4.3.2, the GitHub adapter does not allow bidirectional model synchronization. In our evaluation, we are discussing the impact of this factor on the reactive links and show they can still be used effectively.

### Implementation aspects

To evaluate our proposed solution, we present an implementation used in the evaluation study.

#### DesignSpace as common environment

We begin by outlining the common environment, which is a server that stores all artifacts. For this purpose, we have selected DesignSpace^9^ [27, 51], a versatile platform that facilitates the storage and reasoning of engineering knowledge in a multi-tool and multi-user environment. Any tool that can be connected to DesignSpace can upload its resultant models and their respective metamodel to the server. The data is continuously synchronized and kept consistent, as specified by the engineers.

DesignSpace stores engineering data in a uniform representation, based on a DesignSpace-specific meta-metamodel. Subsequently, the server manages the models stored, enhancing them with additional information where necessary. All the subsequent operations, especially cross-tool and cross-domain operations, are applied at the server level. The meta-metamodel used by DesignSpace is presented on the left side of Fig. 2.

In more detail, DesignSpace stores the model and metamodel information of each domain together. In our context, the metamodel represents the structure of the data, while the model stores the information itself. For example, in the Requirements tool, the metamodel stores the structure of a requirement, while the model stores each requirement specification itself. Storing both the metamodel and model uniformly allows for extensions and connections across the boundaries of domains, as new information can be added to the structure of the data when necessary. Thus, the metamodel is stored in InstanceTypes, which describe the structure of the model elements. Each property in the metamodel is described by a PropertyType. The type of a property can be a generic type, such as string in the previous example, or a reference to a custom InstanceType defined in DesignSpace. After the metamodel is defined through InstanceTypes and PropertyTypes, the model itself is uploaded. Each artifact of the model is stored in an Instance of a specific InstanceType. When an Instance is created, the data of the artifact is stored in the properties defined in the metamodel as property values. If necessary, more properties can be defined dynamically later.

Let us return to our motivational example. In the calculator, this is a parameter, a number stored in a uniform unit of measurement and inserted in any formula needed for calculations. One TechCalc is connected to DesignSpace, the adapter automatically fetches or creates the InstanceType Parameter, with a set of PropertyTypes defining the characteristics of such an artifact (e.g., name as a string, value as a number, unit as an enum value, etc.). Then, the adapter creates the Instance for the maximum piston pressure and assigns the data of this parameter to the Properties of this Instance. Hence, the Property name is set to “maximum piston pressure”, the value is set to 160.0, and the unit is set to “bar”.^10^ Similarly, the requirements tool adapter creates the metamodel for Requirement and stores the artifacts in Instances of this Requirement InstanceType. The requirement Instance corresponding to the maximum piston pressure specification is then enhanced with a dynamically created property that stores the value of 160.0. This property value is then linked to the value of the calculator parameter via reactive links.

#### Uploading data to DesignSpace

To collect the tool-specific data, DesignSpace uses plugin adapters made corresponding with the design goals specified by Sun et al. [100]. Each tool is provided with a custom-built adapter, responsible for translating between the tool’s internal metamodel to the DesignSpace data representation described above. The structure of the tool metamodel is first captured in InstanceTypes and PropertyTypes, before the tool-specific data is uploaded as Instances of these InstanceTypes. This data is automatically uploaded to DesignSpace when the tool is connected to the server for the first time. Then, any change to the data is propagated to the adapter to synchronize the tool and DesignSpace representations. Therefore, if the user changes something in the tool, the adapter sends this to the DesignSpace server. Conversely, if the change originates in DesignSpace, the adapter mirrors it in the original tool.

Our solution is event-based, which means that any change to an artifact results in notifications being sent to the services on the server, i.e., DesignSpace, and the tools connected to it. The communication between the server and the client-side extension is done via gRPC [38]. This communication protocol uses proto requests and responses to send messages across tools, platforms, and interfaces. The messages encode all the information necessary for the receiver to understand and apply the change in their local data.

It must be noted that not all plugins offer two-way communication with the server. While such a bidirectional communication is standard, the GitHub plugin we are using for the Agricultural machines scenario is specifically unidirectional. Namely, any update on the GitHub side will be synchronized and visible in DesignSpace. However, the plugin does not transport the DesignSpace changes back to the GitHub tool. This is a limitation we have to carefully consider when specifying links, especially propagation links, as the GitHub data, much like the TechCalc computation results, is effectively not editable.

#### Collaboration in DesignSpace

As previously mentioned, DesignSpace is focused on a multi-tool (and consequently multi-domain) and multi-user collaboration. Therefore, it offers a variety of collaboration features for different working styles and preferences. In this paper, we are focusing on the default collaboration features.

When using DesignSpace, each user has access to one or more workspaces, which group the artifacts of one tool/domain used by the user. The workspaces are modeled in a tree of inheritance levels, where each workspace can have one parent workspace and several child workspaces. The data in a workspace is private, only visible in this specific workspace, until the user commits their changes, making them visible to the parent workspace. Alternatively, the changes in a parent workspace can be pulled in the child workspace. By default, these committing and updating actions are manually triggered by the user, but they can be automated, in which they are automatically performed when a change is detected.

We find that the intrusiveness of automatic propagation is reduced significantly enough for the benefits of this propagation trigger, e.g., chain propagation, to outweigh its downsides.^11^ Specifically, each collaborator can use a separate workspace, children of a workspace managed by the team manager. The link propagation triggered by a change will be visible to the collaborators of the user who performed the change only after the change is committed, and the collaborators pull the changes from the parent workspace. Each team member can choose the level of intrusiveness they prefer using the automation settings for the committing and updating actions mentioned before. Therefore, in the following, we will consider only automatic propagation links for our evaluations.

#### Implementation of link

**Fig. 5:**
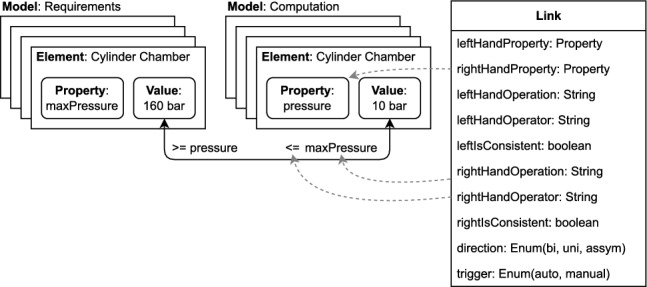
The structure of the link implementation

We have extended the DesignSpace meta-metamodel to include the linking metamodel that specifically targets reactive links for our solution, presented on the right side of Fig. 2. These links are represented by specialized instances that can augment and enrich the metamodels uploaded by the tool adapters. Linking instances have a consistent and unified model for all use cases. The instances are of the type Link and are depicted in Fig. 5. This figure also illustrates the link using our motivating example (discussed in Sect. 2).

As mentioned in the solution description, the aim of the links is to assure granularity by linking property values directly. This is uniquely identified by the instances of Property. Additionally, operator and operation store the link properties, for each of the two ends of the link, together with direction and trigger, which completes the instances. The fields IsConsistent store the current status of each link instance.

Engineers also have the ability to specify simple transformations that need to be applied during propagation. These transformations are executed as the first step when a relevant change occurs. To facilitate this process, we are utilizing ARL [55], which is also used for unit transformations during propagation.

**Fig. 6:**
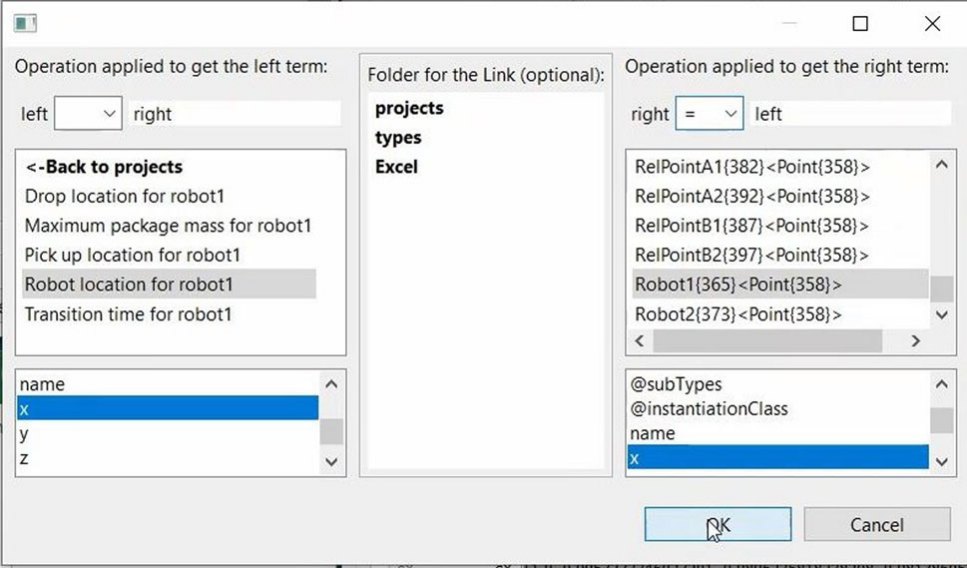
The graphical user interface of the prototype used during the evaluation

To establish a new link, engineers can choose and define the relevant instances and properties to be linked, along with the operators and simple transformations. This information is available for selection via a simple prototype graphical user interface (GUI). A capture of the GUI can be seen in Fig. 6. Once the user makes their selection from the form lists and inserts operations where necessary, the data is transported through a specific gRPC call to the server, where a new link instance is created.

Our solution also features a mechanism to prevent cycles from occurring in the link network. In order to achieve this, we have implemented the algorithm proposed by Tarjan et al. [104], which performs a depth-first search in the network to identify any cycles. We perform this search every time a new link is created. If a cycle is found, we reject the new link and do not allow the cycle to be closed. The prototype we use for the evaluation shows a warning when the link is rejected. While this solution can become quite time-intensive for long link chains, we have found these situations to be very rare in our scenarios.

Additionally, two of our scenarios extensively deal with numeral values. However, we noticed that in most cases, the propagation links between tools with different levels of precision are unidirectional or asymmetrical. This, in combination with the cycle prevention, results in floating point precision differences becoming a somewhat negligible threat to our evaluation.

#### The link reaction process

The execution of actions related to a reactive link is triggered by a change in one of the property values it connects. It goes through the following steps:

1. The change notification is received, and the changed property value is selected from it.
2. The changed value is converted to the standard unit of measurement, if a unit is specified, e.g., a value in *cm* is converted to *m*.
3. The operation is performed if one is specified.
4. The operator is checked.
5. For propagation, the value is converted to the linked property’s unit and then set as the corresponding value unless the values are already equal.
6. For constraint checking, the opposite value is also converted to the standard unit, and then, the two are compared. The result of the comparison is stored in the corresponding consistency flag.

## Results and discussion

After conducting our evaluation study, we were able to assess the feasibility, flexibility, and performance of our solution. To accomplish this, we established reactive links for three engineering scenarios. These links connect all related artifact properties according to requirements and consistency rules. In the following sections, we present each use case we identified, along with practical examples from the scenarios. Additionally, we examine how these links can be applied to various types of property values. Furthermore, we discuss the impact of the links on memory consumption and execution time, taking into account different link distributions. By doing so, we provide a comprehensive analysis of the benefits and potential challenges of incorporating these reactive links into our solution.

### RQ1-Feasibility

In this section, we analyze how our solution works for seven different cases, which shows the feasibility of our reactive links.

#### One-to-one bidirectional propagation

Our first consideration is the most prevalent use case in our scenarios. It involves establishing a connection between two property values and bidirectionally propagating any changes made between them. An example of such a scenario can be found in the hydraulic actuator case. In this instance, the piston cylinder’s diameter must remain consistent between the TechCalc computations and the SolidWorks graphical design. To achieve this, we connected the two properties using a link with an *assignment* operator in *both directions*. The outcome is that any alteration made to the cylinder diameter in either the computational or graphical model is instantaneously propagated to the other end of the link.

#### One-to-one constraint checking

A similar situation occurs when we examine the consistency of the relationship between two property values. In the example detailed in Sect. 2, concerning the hydraulic actuator scenario, the requirement constraint on the piston chamber pressure can be linked to the computation’s value using *comparison operators* on *both directions* of the link. Upon setting up this link, if a developer unintentionally sets the piston chamber pressure too high in the computation, the issue will be flagged in both of the involved tools. Similarly, if the requirement changes, the link will be activated to ensure that the value set in the computation still complies with the requirement.

#### One-to-one unidirectional or asymmetrical linking

In certain situations, the requirement specifies precise values to be utilized in other tools. In such cases, any changes in the specification should be propagated to the other tools employing the corresponding value, while the specifications themselves should not be altered via link propagation. To address this concern, our solution enables the utilization of a *unidirectional link*. The link is established in the same way as a bidirectional propagation link, with the caveat that we only add the assignment operator for the direction of the link that should propagate. The other direction’s operator will be left blank and disregarded. Alternatively, we can use *asymmetrical links* by setting the operator of the other direction to *equals*. In this case, modifications to the specification will be propagated, and any changes in the implementation leading to a constraint violation will prompt the detection of inconsistencies.

A similar situation is captured in the Agricultural machines scenario, where the communication between the hardware and software components of the machines is assured through interfaces with strict naming conventions. As previously mentioned, the GitHub plugin does not push any changes back into GitHub, resulting in the software interfaces being effectively final. Therefore, a link assuring the consistency of the interface attributes naming would use a unidirectional or asymmetrical setup. Any change in the software interface is propagated into the Visio representation, while changes in the Visio representation are checked for consistency against the GitHub data.

#### Links with operations

Additional use cases arise when values need to be slightly altered instead of being propagated directly. Consider the robotic arm scenario, where the minimum arm length is computed using TechCalc based on other worksheet parameters. However, the actual arm length used in the graphical model should be 20% larger than the calculated value. In such instances, the engineers set the links between these corresponding property values similarly to previous cases, but with a notable modification. Each link now specifies the required *operation* to compute the desired value in each direction.

#### One-to-many constraint checking

The previously mentioned use cases primarily focused on individual links. It is important to note, however, that links can interact with one another or be combined to yield more intricate results. An example demonstrating this concept is verifying whether a value falls within a specific interval specified in the requirements.

To address this use case in our solution, we create a pair of comparison links. These two links correspond to the lower and upper bounds of the interval. For example, in the hydraulic actuator scenario, the characteristic coefficient of the piston cylinder computed in a worksheet must remain within a predefined interval for the specific component in use. The engineers use two links to connect this result to the required interval boundaries. Any deviation from the specified range results in an inconsistency being flagged. Furthermore, the developer receives immediate feedback by being promptly notified whether the value is too low or too high.

#### One-to-many propagation

In our solution, links can achieve more intricate use cases with one-to-many propagation paths. An example of this can be observed in the hydraulic actuator scenario, where the length of the piston cylinder is explicitly defined in the requirements and utilized in both the TechCalc and SolidWorks models. To establish this one-to-many connection, we create links between each property in these two models and the corresponding requirement. Consequently, whenever a change is made to the requirement, it is automatically propagated to both models simultaneously, streamlining the process into a single step. This synchronization ensured that all relevant components remained consistently updated with the most recent requirement adjustments.

#### Chain of propagations

Finally, when opting for automatic propagation, we enable the links in our solution to trigger cascading propagation effects. To illustrate this, we consider the same scenario mentioned in the previous section, where the length of the piston has to be synchronized between the requirements, computations, and graphical model. In this case, we establish a link between the requirement and a computation, followed by linking the same computation to a graphical model. The result is a chain of propagations that obtains the same result as the one-to-many linking setup. However, a more practical example encompasses a situation where one link initiates a computation, and the outcome of that computation becomes a component of another link. This specific use case is exemplified in our motivational example. Here, a link connects the threshold value of the piston chamber pressure between the requirements and the computation, which is used to calculate the piston cylinder diameter. Subsequently, this resulting diameter is linked to the corresponding graphical design artifact. Let us consider a scenario where the client decides to select a different off-the-shelf component with specific threshold pressure requirements and wishes to determine whether it can safely be used in the system. In this case, the specified threshold in the requirements is adjusted to the new value, triggering an instantaneous propagation to TechCalc. The TechCalc worksheet is automatically recomputed, generating a new cylinder diameter that is propagated through the links to SolidWorks. This entire process occurs nearly instantaneously, eliminating the potential for human error that may arise when manually synchronizing data.
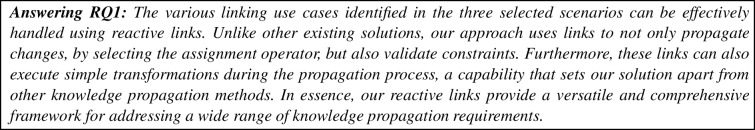


### RQ2-Flexibility

Two of our scenarios, the Hydraulic actuator and the Robotic arm, are highly based on numerical values. Meanwhile, the most prominent data types in the Agricultural machines scenario are strings and collections. In this RQ, we focus on the analysis of whether our solution can be applied in the context of the varied data types captured in these scenarios.

Firstly, different variations of links connecting numerical data were captured, as described in the results of the RQ above. The reactive links of our solution offer a wide variety of operators aimed at numerical comparison and propagation. However, the links can be extended with new operators that are designed to evaluate the consistency of different data types. For example, for the set of attributes representing the types and names of the interface fields in the Agricultural machines scenario, we have extended our solution with collection-specific operators. More specifically, we add the operator *contains* to check that a string property value is included in the values of a collection-typed property. With this, we set a link between each attribute in the UML interfaces and the collection of attributes in the GitHub file storing the equivalent interface. This process can be automated, due to the strict naming conventions used by the industry partners, resulting in such a link being automatically added to each new attribute in the interface. Therefore, the inconsistency will be flagged when a new attribute is specified in the Visio interface but not yet added to the GitHub file.

Not all the reactive link operators are restricted to certain data types. While the comparison operators, such as *less than* (<), only accept numerical property values, the *equality* ($==$) or *assignment* ($=$) operators accept any property values as long as the types match and can be represented in the DesignSpace meta-metamodel. The use of these operators is described in Sect. 5.1.3, when linking the names of two equivalent interface elements in the software and hardware component models. Due to the limitations of the GitHub plugin, these links can either be unidirectional or asymmetrical. The asymmetrical case is defined using the assignment operator in the GitHub-to-Visio direction, and the equality operator in the opposite direction. The main limitation, in this case, is that links are designed for simple links. This means that they may not be suitable for extensive navigation, such as collecting data from multiple property values or across multiple trace links, and complex transformations, such as computing or aggregating values based on multiple properties. It must also be noted that the prototype does not support implicit type casting due to an implementation decision. This limitation is specific to the prototype itself, the reactive links can be implemented to allow implicit type casting.

Alternatively, we can directly link the two sets of attributes through a propagation link. In this case, if a new element is added to a GitHub interface, the change is propagated and can be mirrored by the Visio plugin in the UML representation. Similarly to the cases captured in Sect. 5.1.3, these links can either be unidirectional or asymmetrical.

Moreover, the type of the value is not always the same in both of the linked properties. Going back to the Robotic Arm scenario, we used the robot arm length in the code of the software which runs on the robotic arm. The arm length is a *floating point* value, while the IntelliJ adapter stores all the data in *text* format. Therefore, the link toward the code property will transform the value to a string through an added operation. The opposite direction can have an operation that parses the string back to a number.
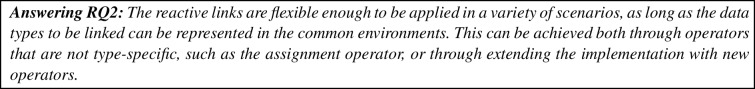


### RQ3-Performance

The performance of the system is evaluated from three perspectives. On the one hand, we are discussing the effort necessary for setting and maintaining links from the user’s perspective, in comparison with manually propagating changes. Next, we analyze the impact of these additional linking artifacts on memory usage in each engineering scenario. Finally, we evaluate the most common use cases of the reactive links in our engineering scenarios, in terms of run time performance.

To begin with, we must address the overhead of setting up the models and links, as this can appear to be an additional step in the development process. However, as described in Sect. 3.10, the dependencies affected by a change have to be identified and maintained even in the absence of our links. After they are identified, ideally early in the development process, these dependencies have to be documented in preparation for future changes that may touch the same artifacts. Then, after every new set of changes, the affected dependencies have to be checked for consistency and the impact of the changes has to be manually propagated.

Instead, if a link is set up immediately when a dependency is identified, the maintenance of consistency across this dependency will be automatically ensured with minimal additional user interaction. Moreover, setting these links only requires the completion of a simple connection form in the prototypical GUI provided. The engineer must select the artifacts to be connected, and the name of the properties holding the dependent values from a list. Then, using a drop-down menu, they can select the operators that define the link behavior. Optionally, engineers can specify the operation, or simple transformation, in the text field provided for each direction of the link. Once the form is complete, the user submits the inserted data to DesignSpace at the push of a button.

After creation, the links are automatically maintained by DesignSpace and activated whenever a change needs to be propagated. As mentioned previously, each link is an instance of the LinkType instance type, storing the identification of the linked property values and the operators and operations. As a result, a link is an instance with ten simple properties, which are either references to other instances, or string values. In the context of our engineering scenarios, the addition of links is negligible in terms of memory usage as compared to the number of instances and properties necessary for storing the model data itself.

Based on the engineering scenarios we considered, which vary both in terms of domains and in terms of size, the links needed to cover the cross-tool dependencies in our projects are limited. The distribution of links in comparison with the total number of artifacts in each scenario can be seen in Table 6. For the Hydraulic actuator and Robotic arm scenarios, an average of $\approx$6% of the number of artifacts in the system is taken by the links. Even in the case of the Agricultural machines, in which the collaboration between the software and hardware parts of the system requires exact matches between several property values, the links necessary to ensure the consistency of the system only represent $\approx$16% of the number of instances stored in DesignSpace as part of this scenario. Additionally, each of these links has a very lightweight model, which comes in contrast to many other artifacts generated by the tools used during development. As a result, the impact of the links, in terms of the information stored as property values in the link artifacts, is lessened as the systems under development grow.

**Table 6:** The number of artifacts and properties added by the reactive links in our evaluation scenarios

Scenario	# Links	# Link properties	% of Instances	% of Properties
Agricultural machines	28	280	16.0	17.5
Hydraulic actuator	17	170	5.8	9.9
Robotic arm	53	530	5.4	3.5

In terms of run time, we have considered two cases that are prevalent in our engineering scenarios. The first case evaluated is the *one-to-one bidirectional propagation*. After ten executions, we determined that the propagation across this type of link takes 0.045 milliseconds, which is already beyond the reaction time of any developer. This execution time does not differ significantly between floating point values, text values, and set values. We then considered the added impact of an *operation*, so we selected the case in which the robot arm length has to be propagated from the computation to the Java code, and converted to string. This propagation took, on average, 0.053 milliseconds. The difference between this and the previous case is the application of the Java toString() method. The Agricultural Machines scenario introduces the “contains” set operator. However, the implementation of sets in our context is strongly based on the Java HashSet representation, for which the contains method has O(1) time complexity. As a result, the evaluation time of this operator is similar to that of the propagation operator.

Another common use case is the *chain propagation*. We have chosen to evaluate the scenario in which the piston cylinder diameter is specified in the requirements, linked to the computation, and then, the same computation parameter is linked to the graphical design. This use case allows us to evaluate chain propagation without the interference of tool operations. This process takes 0.079 milliseconds on average.

**Fig. 7:**
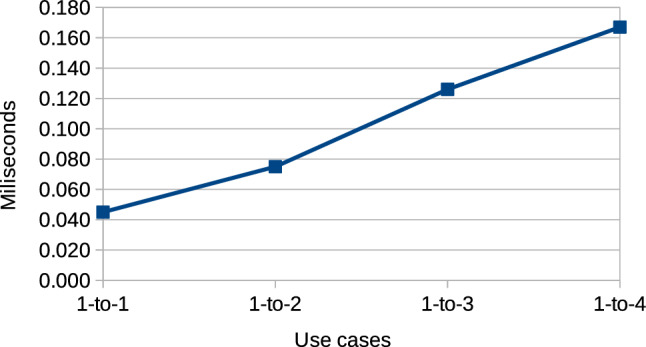
Execution time to propagate 1-to-*n* links, varying the value of n from 1 to 4


The evaluations so far only consider situations with small numbers of links. To evaluate how the execution of links scale, we considered the *one-to-n* use case and timed it for different values of *n*, which is presented in Fig. 7. The one-to-one propagation gives the same result as mentioned before. For *n* between 2 and 4, the propagation time *increased linearly*, as expected, with around 0.04 milliseconds for each additional link.

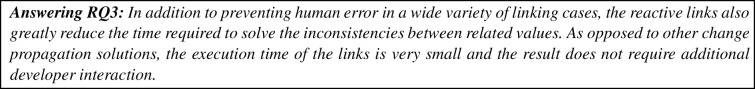


## Limitations and threats to validity

In this section, we discuss the limitations of our solution and the internal and external threats of our study that evaluates our solution.

### Limitations of our solution

One limitation of our solution arises from precision level differences in numeric values. While not a significant issue in our evaluation scenarios as a result of the types of links needed, this choice should be reconsidered before the prototype can be released to a real-world environment. For this reconsideration, one can consider the approaches discussed in Sect. 3.8 in accordance with the needs of the targeted clients.

Another limitation is that our implementation does not have an efficient approach to circular linking, and the prototype has not been thoroughly tested in terms of detecting circular links. The approach we have chosen to handle this possible issue is markedly inefficient. While the performance of the link triggering is not affected by the choice of circularity management, the time and resources necessary to create a link are both heavily influenced. We suggest a number of possible improvements and alternative implementations in Sect. 3.9.

Lastly, our prototype uses DesignSpace as a common environment, which provides good support for cross-domain linking and collaborative development. However, in order to connect and upload the models from a tool to DesignSpace, it is necessary that an adapter for that tool is available. This results in a limitation on the tools that can be used. The adapters are designed based on transparent adaptation [100], which enables the creation of an adapter for a wide array of different tools but comes with a set of limitations in itself. Additionally, these adapters have to be created and maintained for each tool used. A new tool or a new version of an existing tool will likely require a new adapter or extensive changes to the existing adapter.

### Internal threats

There are some internal threat issues to be considered, especially regarding the evaluation method. We considered three scenarios for the evaluation, but only the modeling part of the projects. We did not have the possibility of capturing the discussions of engineers or industry settings to accurately compare the results with the process used in the industry.

Another internal threat is that the decisions taken during the implementation of our solution might have influenced the results, mostly the execution time. However, we believe this is a minor threat, as the different use cases in the performance evaluation considered the same implementation, only varying the number of links.

Regarding the analysis of the result, we have limited input from industry users using our solution, and therefore, we could not accurately evaluate the usefulness of the solution in a distributed environment. However, we assume that there is no difference between links and other artifacts from a collaborative perspective.

### External threats

Our work is also susceptible to external threats in terms of the generalization of the results. It is not possible to assert that other scenarios or systems possess the same features as those in our evaluation. Nonetheless, we have identified a considerable number of corner cases and intricate links within our scenarios. Testing the solution on a larger system would undoubtedly reveal additional challenges, as well as provide us with an opportunity to further assess the solution’s performance.

## Related work

The synchronization of changes has been a persistent challenge that developers have faced, especially in collaborative engineering. Various approaches have been proposed in the literature to tackle this problem, each focusing on specific aspects while overlooking others. This section provides an overview of the most common solutions proposed in the literature.

### Traceability management

The first aspect to be discussed is how to maintain connections between logically related artifacts from different models or tools. Klespitz et al. [60] mention that duplicate information in multiple locations and artifacts requires either unnecessary and tedious manual maintenance, or an automatic solution that performs the equivalent without increased involvement from the engineers. Then, the authors propose a solution based on OSLC links for cross-tool traceability. The OSLC protocol is adopted by multiple tool suites available in practice, such as IBM Doors [56], allowing the engineers to connect artifacts developed and stored in different tools. Other solutions focus on traceability links with different metamodels. As traceability is most commonly studied in requirements engineering [93], multiple solutions focus on requirements traceability [68, 73] using features already available in requirements management tools, such as ReqIF [30]. Ontologies [17, 18], topic modeling [8], or graph networks [93] are proposed to adapt traceability in the context of model-driven development. Similarly, artifact management systems like ADAMS [25] store the artifacts in a uniform representation that supports traceability.

Linsbauer et al. [66] proposed another solution that uses trace links. They recover and maintain the variability of a product line system by tracing the dependencies between procedures and the abstract syntax tree generated from the variant execution. Trace links can also store additional information about the system, which can be used for further processing. For example, the Dapper platform [96] uses trace links to capture communication within a distributed system. Asuncion et al. [7] suggest using the Federated Information-based Design Environment to store trace links between components and enrich them with additional information about the system’s construction and status.

Further, some traceability approaches focus on incremental traceability management between artifacts contained in heterogeneous models [15, 94, 95], similarly to those targeted by our solution. A feature necessary in each heterogeneous traceability management solution is ensuring a uniform or compatible representation for all the artifacts to be traced. Heisig et al. [50] address this issue through the use of MOF, providing a unified modeling basis for their solution. Similar solutions use Ecor [5] for the same purposes. Indeed, this is the basis for tools such as Eclipse CAPRA [31] and YAKINDU [57], which allow traces between any types of artifacts compatible with the EMF framework available in the Eclipse environment. Additionally, different approaches enhance the traceability support systems with automatic trace maintenance [18, 25, 85] and navigability for consistency checking [93] and further trace analysis [73]. Ratiu et al. [86] propose a flexible traceability support solution similar to Eclipse CAPRA, with an added level of extensibility at runtime.

These solutions demonstrate the usefulness of trace links in maintaining connections between logically related artifacts from different models or tools. However, despite the number and variety of available traceability approaches, a common feature among all of them is the relatively coarse granularity. Trace links allow the connection of artifacts, but cannot be used to specifically link two artifact properties without extensive remodeling of the data model, or potentially significant redundancy. To this end, the reactive links provide a level of granularity more fit for property value change propagation and inconsistency detection, while trace links can be used to further document and maintain the consistency at artifact level.

### Maintaining consistency across tools

Ensuring data consistency is another crucial aspect of synchronization that needs to be addressed, both within a model and across different related models. While most development tools provide functionalities to ensure consistency within models, it becomes challenging to achieve consistency when multiple developers work on the same project.

Alvaro et al. [4] propose various solutions to address this issue at multiple levels of the system, such as storage level, runtime, or compile time via language-encoded annotations. Some solutions integrate consistency checking with traceability to cross model and tool boundaries. For instance, Adersberger and Philippsen [2] propose a UML extension that automatically checks for consistency between architecture specifications and implementation code. Similarly, Tröls et al. [107] combine an artifact management platform with trace matrix representations and consistency rules written in Object Constraint Language (OCL [112]) to ensure consistency across tools and models.

When traces are not available, solutions for maintaining consistency often restrict the set of tools and languages that can be used by engineers. Although adding traceability features can help alleviate this issue, it can also lead to a lack of granularity, as previously discussed. This approach is appropriate for certain cases. For example, consistency rules can be used to check that all requirements are prioritized and categorized. In combination with traceability, they can ensure that each requirement is implemented and tested. However, even with traces available, consistency checking, as described in the approaches above, is too coarse and general to check a situation like our motivational example: one property of one single requirement must correspond to one property on one specific model element. Reactive links can provide the required level of granularity for this use case, while consistency rules can be specified in parallel for coarser-grained constraints.

### Change propagation

Ensuring the consistency of changes across a software-intensive system is a fundamental aspect of synchronizing models, particularly in the context of distributed and collaborative model-driven engineering. However, this often proves to be a challenging task, with many change propagation solutions requiring extensive user involvement, which can increase the risk of failure and result in slower and less efficient propagation. To address this issue, different approaches focus on supporting the manual change propagation process by providing recommendations on optimal propagation paths or predicting the impact of change propagation.

Identifying the optimal change propagation path provides guidance to engineers who manually address the impact of a change. One proposed solution by Tang et al. [102] utilizes a matrix that stores dependencies between the components of a system to reconstruct all possible propagation paths starting from a modified component. The algorithm suggests the shortest and most efficient path to the user. Another approach by Ullah et al. [109] formulates a mathematical model that captures the propagation impact and utilizes it to parse a design structure matrix, resulting in the path that provides the best coverage of the risk. While these solutions have advantages such as preventing accidental omissions, they may also introduce significant overhead to each change propagation event.

Predicting the impact of change propagation offers the advantage of not triggering with every change in the system. For instance, Pasqual et al. [77] proposed a solution that utilizes a multi-level network constructed from the history of the system. The network encompasses the development team organization, the product structure, and the change history, as well as the relationships between these layers. Similarly, Hamraz et al. [46] use a network that contains the functional, behavioral, and structural components of the system. Another solution, suggested by Ahmad et al. [3], proposes a network that traces the connection between requirements, function, and component structure, and the detailed design process. These matrices become a foundation for change propagation algorithms and heuristics.

Change propagation solutions have practical applications and are particularly useful for exploring different component linking possibilities. However, these solutions often require extensive user involvement to ensure changes are propagated correctly. In contrast, once set, reactive links automatically propagate changes or check fine-grained constraints every time a relevant update occurs. While manual propagation may be necessary in certain cases, such as when complex reasoning is required, in most practical scenarios, the automatic propagation provided by the reactive links is both effective and efficient. However, when complex reasoning is needed, or changes at a level the reactive links cannot provide for, change propagation solutions can help engineers ensure that the propagation is complete.

### Automatic knowledge propagation

The relationships between system components can be represented in various ways, not just through trace links. For instance, Jelly [70], a GUI design tool, employs a semantic network to identify and maintain the correspondence between equivalent components in different graphical interface frameworks. Whenever a component undergoes a change, Jelly replicates the changes in similar components across all frameworks. Alternatively, El-Khoury et al. [32] propose a solution that relies on a specific framework for the development and maintenance of data, allowing users to specify the domain description, dependencies between resources, data allocation, and tool structure.

While these solutions offer advantages in terms of change propagation, consistency across tools, and linking of related components, they both have a significant drawback: they severely limit the selection of tools and domains that can be used during development. For instance, Jelly works only on specific GUI development tools, while the system suggested by El-Khoury et al. is not implemented in all tools. It is not practical for engineers in the industry to learn and adopt new sets of tools for each project and problem they encounter during development.

In practice, one approach that has been widely utilized involves leveraging databases and their associated conditions. This approach allows for the detection, classification, handling, and documentation of inconsistencies within the system. For instance, Easterbrook et al. [29] and Finkelstein et al. [36] propose solutions that utilize consistent overlapping partitions of the data to achieve these goals. Notably, these partitioning techniques are designed to be independent of specific software development methods or toolsets. Similarly, Grundy et al. [43] enable the specification of various system views that can be linked and synchronized. Reinert and Ritter [87], on the other hand, employ Event-Condition-Action specifications to establish links between different model views.

These solutions have been developed and refined over a significant period of time, both in research and industry, and have yielded valuable improvements and outcomes. However, one notable drawback is their reliance on a shared database for storing model elements. While this approach may be suitable for certain scenarios, such as single-domain solutions or during the requirement specification process, it falls short in cases involving multiple domains and tools. Attempting to consolidate such diverse environments into a single database can lead to a substantial loss of information.

### Multiview development systems management

Different approaches focus on ensuring the consistency of the data contained in different views of complex models. The issues arising from managing such multi-view models have been widely explored [71], resulting in multiple frameworks [35, 53, 76] or database solutions [88] aimed at managing dependencies between different viewpoints. These dependencies are modeled as links between different elements of these views [36, 75], through which the developers can observe the consistency of their system and define actions to be taken when inconsistencies appear.

While these approaches offer a variety of inconsistency management solutions, varying from change propagation to more complex logical rule definitions, they are based on heterogeneous views of a model, rather than heterogeneous models that represent different sections of the system. Therefore, these approaches require a single, consistent metamodel, each view customizing what areas of the model are presented and managed by the user. In multi-tool and multi-user development, this requires a harmonization of the different metamodels of each tool and domain, which may also lead to loss of information that was part of these different metamodels.

### Model-to-model transformations

One of the options for ensuring the consistency of systems formed by multiple different models is to propagate changes across the dependencies between these models. An approach toward achieving this is defining a model-to-model transformation, which would automatically generate the equivalent representation of the data from one model into another. A variety of tools focused on this method already exist, as described by Kahani et al. [58].

For these transformations, one needs to define the correspondence between a source model and a target model, based on which the target model is automatically generated from the source. Depending on the approach, the two models can have homogeneous or heterogeneous models. In the case of heterogeneous models, the transformation rules define how the source model artifacts are to be translated into the target metamodel. In order to do so, the source and target metamodels must both be specified, followed by an explicit translation or mapping of the artifact information between the two metamodels. While some transformation solutions require a common ancestor between the metamodels, it is not uncommon that this is not necessary. Often, the actual information of the model is abstracted away, and all the logic behind the transformation is done at a metamodel level. The transformations can be incremental [41], in which case the transformation rules are applied only for the changes in the source model that need to be propagated, leaving the rest of the target model unchanged. Additionally, the transformations can be bidirectional [20, 28, 80], in which case changes occurring in either of the models will be propagated. In most cases, after the transformation is defined, the source model is transformed into a target model with limited traceability as to where each change came from. Additionally, as the transformation is defined at the metamodel level, it can be difficult to identify and pinpoint exactly the property values that need to be synchronized with very fine granularity.

Model-to-model transformations, especially when bidirectional, incremental, and managing heterogeneous models, offer a scalable and feasible approach to maintaining cross-domain change propagation. However, they require a mapping between the metamodels of the two domains, the definition of which can become challenging. This is especially the case for situations targeted by reactive links, where instead of mapping all the information of an artifact type from the source metamodel to the target metamodel, we are only concerned with propagating specific values from specific artifact properties. When the number of dependencies between models is significant, these approaches are more advantageous than managing many individual links. However, similarly to the consistency checking approaches, the number and specificity of our propagation needs result in the mapping needed for the transformation becoming unmanageably complex. Additionally, the dependencies between artifact properties are not regular (e.g., each requirement has one property that links to one model element property) but rather random (e.g., only this requirement has a pressure specification, and one more requirement has an arm length specification to be linked with the graphical design tool). In this case, the mapping of the metamodels may need to be updated every time a new dependency is identified. Thus, in these use cases, defining autonomous, reactive links achieves the same goals as model-to-model transformations without the need to define and maintain metamodel-dependent transformation rules.

## Conclusion

We proposed and evaluated a novel solution to ensure consistency and enforce constraints among artifacts in multi-tool/domains and multi-users scenarios when engineering complex software-intensive systems. Our approach involves creating links that connect artifacts in a highly granular network of related property values, which react automatically to changes in any of their connected values. This reaction can be customized to either propagate the change throughout the network or alert the user of any constraint violations introduced.

The evaluation of our solution demonstrated not only the feasibility of the proposed links in terms of the covered use cases, but also the potential for significant improvements in the development process. The reactive links are highly adaptable and can be utilized in a range of scenarios, as long as the data types involved can be represented within the common environments. Moreover, the introduction of faults can be quickly detected with the links, and in many cases, inconsistencies can be resolved faster and more accurately through propagation across the links than through manual intervention by developers.

While our proposed solution has shown promising results, there are still some limitations and areas that need further exploration. To expand our understanding of the system, we plan to conduct tests on larger systems and in real-world development environments. By gathering feedback from engineers, we can identify areas for improvement and refine the solution to better meet their needs.

Although our solution provides a strong foundation for linking artifacts, there is still room for further development and customization to accommodate specific use cases. For example, our current implementation lacks the ability to handle precision differences, which can be a significant issue in complex engineering scenarios. Also, for a better user experience regarding cyclic linking, the user interface can be extended to provide more information on the cycles found, including the properties involved in the cycle. Addressing these limitations would significantly enhance the usability and applicability of our proposed solution, which can be investigated more thoroughly in future research.

## Data Availability

Due to copyright concerns around the DesignSpace plugins and evaluation scenarios, we cannot make the data and full prototype openly available. However, we provide a replication package for a limited part of the evaluation. Additionally, we provide the binary version of the solution used in our evaluation, as well as some guidance toward creating custom plugins. These documents are available at: https://doi.org/10.5281/zenodo.11104978.

## Footnotes

1 From Linz Center of Mechatronics GmbH, Austria (https://www.lcm.at/en/) and Flanders Make, Belgium (https://www.flandersmake.be)

2 A video with the demonstration of this scenario is available at: https://youtu.be/4Z9qy9PM2PY.

3 A tool developed within our institute.

4 A tool developed within Linz Center of Mechatronics GmbH: https://www.lcm.at/project/techcalc/

5 https://www.solidworks.com/

6 A video with the demonstration of this scenario is available at: https://youtu.be/gdqROocDq30.

7 https://www.microsoft.com/microsoft-365/excel

8 https://www.jetbrains.com/idea/

9 More details available at: https://isse.jku.at/designspace

10 DesignSpace can store physical values with their associated unit of measurement and perform simple conversions between units. This feature allows the accurate propagation of values between two properties with different associated units of measurement.

11 A demonstrative video of the reactive links in a collaborative engineering scenario is available at: https://www.youtube.com/watch?v=hlfo9DOCCos

## References

[1] Abello, J., Ham, F.V., Krishnan, N.: Ask-graphview: a large scale graph visualization system. IEEE Trans. Visual Comput. Graphics **12**(5), 669–676 (2006) 10.1109/TVCG.2006.120 17080786

[2] Adersberger, J., Philippsen, M.: Reflexml: Uml-based architecture-to-code traceability and consistency checking. In: Crnkovic, I., Gruhn, V., Book, M. (eds.) Software Architecture, pp. 344–359. Springer, Heidelberg (2011)

[3] Ahmad, N., Wynn, D.C., Clarkson, P.J.: Change impact on a product and its redesign process: a tool for knowledge capture and reuse. Res. Eng. Des. **24**(3), 219–244 (2013)

[4] Alvaro, P., Bailis, P., Conway, N., Hellerstein, J.M.: Consistency without borders. In: 4th Annual Symposium on Cloud Computing, Association for Computing Machinery, New York, NY, USA, SOCC ’13 (2013)

[5] Anquetil, N., Kulesza, U., Mitschke, R., Moreira, A., Royer, J.C., Rummler, A., Sousa, A.: A model-driven traceability framework for software product lines. Softw. Syst. Model. **9**, 427–451 (2010)

[6] Anzt, H., Lukarski, D., Tomov, S., Dongarra, J.: Self-adaptive multiprecision preconditioners on multicore and manycore architectures. In: High Performance Computing for Computational Science–VECPAR 2014: 11th International Conference, Eugene, OR, USA, June 30–July 3, 2014, Revised Selected Papers 11, Springer, pp. 115–123 (2015)

[7] Asuncion, H.U., Taylor, R.N.: Capturing custom link semantics among heterogeneous artifacts and tools. In: 2009 ICSE Workshop on Traceability in Emerging Forms of Software Engineering, IEEE, pp. 1–5 (2009)

[8] Asuncion, H.U., Asuncion, A.U., Taylor, R.N.: Software traceability with topic modeling. In: Proceedings of the 32nd ACM/IEEE international conference on Software Engineering-Volume 1, pp. 95–104 (2010)

[9] Auber, D.: Tulip-a huge graph visualization framework. In: Graph drawing software, pp. 105–126 (2004)

[10] Beck, F., Burch, M., Diehl, S., Weiskopf, D.: A taxonomy and survey of dynamic graph visualization. Comput. Gr. **36**, 133–159 (2017) 10.1109/TVCG.2011.226 22034355

[11] Bender, M.A., Fineman, J.T., Gilbert, S., Tarjan, R.E.: A new approach to incremental cycle detection and related problems. ACM Trans. Algorithms **12**(2), 1–22 (2015)

[12] Bennett, C., Ryall, J., Spalteholz, L., Gooch, A.: The aesthetics of graph visualization. In: CAe, pp. 57–64 (2007)

[13] Bernstein, A., Chechi, S.: Incremental topological sort and cycle detection in expected total time. In: Proceedings of the Twenty-Ninth Annual ACM-SIAM Symposium on Discrete Algorithms, SIAM, pp. 21–34 (2018)

[14] Bernstein, A., Dudeja, A., Pettie, S.: Incremental scc maintenance in sparse graphs. In: 29th Annual European Symposium on Algorithms (ESA 2021) (2021)

[15] Beyhl, T., Hebig, R., Giese, H.: A model management framework for maintaining traceability links. Software Engineering 2013-Workshopband (2013)

[16] Borrmann, A., Hyvärinen, J., Rank, E.: Spatial constraints in collaborative design processes. In: International Conference on Intelligent Computing in Engineering (ICE09), pp. 1–8 (2009)

[17] Bougdira, A., Ahaitouf, A., Akharraz, I.: Conceptual framework for general traceability solution: description and bases. J. Model. Manag. **15**(2), 509–530 (2020)

[18] Bougdira, A., Akharraz, I., Ahaitouf, A.: A traceability proposal for industry 4.0. J. Ambient. Intell. Humaniz. Comput. **11**, 3355–3369 (2020)

[19] Brings, J., Daun, M., Bandyszak, T., Stricker, V., Weyer, T., Mirzaei, E., Neumann, M., Zernickel, J.S.: Model-based documentation of dynamicity constraints for collaborative cyber-physical system architectures: findings from an industrial case study. J. Syst. Architect. **97**, 153–167 (2019)

[20] Buchmann, T., Bank, M., Westfechtel, B.: Bxtenddsl: a layered framework for bidirectional model transformations combining a declarative and an imperative language. J. Syst. Softw. **189**, 111288 (2022)

[21] Buttari, A., Dongarra, J., Langou, J., Langou, J., Luszczek, P., Kurzak, J.: Mixed precision iterative refinement techniques for the solution of dense linear systems. Int. J. High Perform. Comput. Appl. **21**(4), 457–466 (2007)

[22] Byun, J., Rhew, S., Hwang, M., Sugumara, V., Park, S., Park, S.: Metrics for measuring the consistencies of requirements with objectives and constraints. Requir. Eng. **19**(1), 89–104 (2014)

[23] Cleland-Huang, J., Gotel, O.C.Z., Huffman Hayes, J., Mäder, P., Zisman, A.: Software traceability: Trends and future directions. In: Future of Software Engineering Proceedings, FOSE 2014, pp. 55–69. Association for Computing Machinery, New York, NY, USA (2014)

[24] Dasanayake, S., Aaramaa, S., Markkula, J., Oivo, M.: Impact of requirements volatility on software architecture: How do software teams keep up with ever-changing requirements? J. Softw. Evol. Process **31**(6), e2160 (2019)

[25] De Lucia, A., Oliveto, R., Tortora, G.: Adams re-trace. In: ACM/IEEE 30th International Conference on Software Engineering, IEEE, pp. 839–842 (2008)

[26] Dekker, T.J.: A floating-point technique for extending the available precision. Numer. Math. **18**(3), 224–242 (1971)

[27] Demuth, A., Riedl-Ehrenleitner, M., Nöhrer, A., Hehenberger, P., Zeman, K., Egyed, A.: Designspace: An infrastructure for multi-user/multi-tool engineering. In: 30th Annual ACM Symposium on Applied Computing, ACM, pp. 1486–1491 (2015)

[28] Diskin, Z., Xiong, Y., Czarnecki, K., Ehrig, H., Hermann, F., Orejas, F.: From state-to delta-based bidirectional model transformations: The symmetric case. In: Model Driven Engineering Languages and Systems: 14th International Conference, MODELS 2011, Wellington, New Zealand, October 16-21, 2011. Proceedings 14, Springer, pp. 304–318 (2011)

[29] Easterbrook, S., Nuseibeh, B.: Using viewpoints for inconsistency management. Softw. Eng. J. **11**(1), 31–43 (1996)

[30] Ebert, C., Jastram, M.: Reqif: seamless requirements interchange format between business partners. IEEE Softw. **29**(5), 82–87 (2012)

[31] Eclipse: Eclipse capra. https://projects.eclipse.org/projects/modeling.capra (2023)

[32] El-Khoury, J., Gurdur, D., Loiret, F., Törngren, M., Da Zhang, M.N.: Modelling support for a linked data approach to tool interoperability. ALLDATA **2016**, 51 (2016)

[33] Elmqvist, N., Do, T.N., Goodell, H., Henry, N., Fekete, J.D.: Zame: Interactive large-scale graph visualization. In: 2008 IEEE Pacific visualization symposium, IEEE, pp. 215–222 (2008)

[34] Ferreira, S., Collofello, J., Shunk, D., Mackulak, G.: Understanding the effects of requirements volatility in software engineering by using analytical modeling and software process simulation. J. Syst. Softw. **82**(10), 1568–1577 (2009)

[35] Finkelstein, A., Kramer, J., Nuseibeh, B., Finkelstein, L., Goedicke, M.: Viewpoints: a framework for integrating multiple perspectives in system development. Int. J. Softw. Eng. Knowl. Eng. **2**(01), 31–57 (1992)

[36] Finkelstein, A., Gabbay, D., Hunter, A., Kramer, J., Nuseibeh, B.: Inconsistency handling in multiperspective specifications. IEEE Trans. Softw. Eng. **20**(8), 569–578 (1994)

[37] Firesmith, D.: Engineering safety requirements, safety constraints, and safety-critical requirements. J. Obj. Technol. **3**(3), 27–42 (2004)

[38] Foundation, CNC.: grpc. https://grpc.io/, Accessed 19 Sep 2021 (2022)

[39] France, R., Rumpe, B.: Model-driven development of complex software: a research roadmap. In: Future of Software Engineering (FOSE), pp. 37–54 (2007)

[40] Franzago, M., Ruscio, D.D., Malavolta, I., Muccini, H.: Collaborative model-driven software engineering: a classification framework and a research map. IEEE Trans. Softw. Eng. **44**(12), 1146–1175 (2018)

[41] Ghorbani, M., Sharbaf, M., Zamani, B., et al.: Incremental model transformation with epsilon in model-driven engineering. Acta Informatica Pragensia **11**(2), 179–204 (2022)

[42] Goubault, E.: Static analyses of the precision of floating-point operations. In: International Static Analysis Symposium, Springer, pp. 234–259 (2001)

[43] Grundy, J.C., Hosking, J.G., Mugridge, W.B.: Supporting flexible consistency management via discrete change description propagation. Softw. Pract. Exp. **26**(9), 1053–1083 (1996)

[44] Hadlak, S., Schumann, H., Schulz, H.J.: A survey of multi-faceted graph visualization. In: EuroVis (STARs), pp. 1–20 (2015)

[45] Haeupler, B., Kavitha, T., Mathew, R., Sen, S., Tarjan, R.E.: Incremental cycle detection, topological ordering, and strong component maintenance. ACM Trans. Algorithms **8**(1), 1–33 (2012)

[46] Hamraz, B., Caldwell, N.H., Ridgman, T.W., Clarkson, P.J.: Fbs linkage ontology and technique to support engineering change management. Res. Eng. Des. **26**(1), 3–35 (2015)

[47] Harary, F., Norman, R.Z., Cartwright, D.: Structural Models: An Introduction to the Theory of Directed Graphs, 4th edn. Wiley, New York (1965)

[48] Hassan, A., Holt, R.: Predicting change propagation in software systems. In: 20th IEEE International Conference on Software Maintenance, 2004. Proceedings., pp. 284–293 (2004)

[49] Hecht, M.S., Ullman, J.D.: Characterizations of reducible flow graphs. J. ACM **21**(3), 367–375 (1974)

[50] Heisig, P., Steghöfer, J.P., Brink, C., Sachweh, S.: A generic traceability metamodel for enabling unified end-to-end traceability in software product lines. In: Proceedings of the 34th ACM/SIGAPP Symposium on Applied Computing, pp. 2344–2353 (2019)

[51] Herac, E., Assunçãao, W., Marchezan, L., Haas, R., Egyed, A.: A flexible operation-based infrastructure for collaborative model-driven engineering. J. Obj. Technol. **22**(2), 1–14 (2023)

[52] Herman, I., Melançon, G., Marshall, M.S.: Graph visualization and navigation in information visualization: a survey. IEEE Trans. Visual Comput. Gr. **6**(1), 24–43 (2000)

[53] Herzig, S.J., Qamar, A., Paredis, C.J.: An approach to identifying inconsistencies in model-based systems engineering. Procedia Comput. Sci. **28**, 354–362 (2014)

[54] Hull, E., Jackson, K., Dick, J.: Requirements Engineering in the Solution Domain, pp. 109–129. Springer, London (2005)

[55] IBM: Advanced rule language (arl). https://www.ibm.com/docs/en/dbaoc?topic=languages-advanced-rule-language-arl, Accessed 06 Sep 2021 (2022)

[56] IBM: Ibm doors. https://www.ibm.com/products/requirements-management (2023)

[57] itemis: Yakindu. https://www.itemis.com/en/products/itemis-create/ (2023)

[58] Kahani, N., Bagherzadeh, M., Cordy, J.R., Dingel, J., Varró, D.: Survey and classification of model transformation tools. Softw. Syst. Model. **18**, 2361–2397 (2019)

[59] Kamrani, A.K., Nasr, E.S.A.: Collaborative Engineering. Springer, Berlin (2008)

[60] Klespitz, J., Bíró, M., Kovács, L.: Enhanced traceability and consistency with augmented lifecycle space. In: 2016 IEEE 20th Jubilee International Conference on Intelligent Engineering Systems (INES), IEEE, pp. 207–212 (2016)

[61] Knuth, D.E., Szwarcfiter, J.L.: A structured program to generate all topological sorting arrangements. Inf. Process. Lett. **2**(6), 153–157 (1974)

[62] Konôpka, M., Bieliková, M.: Software developer activity as a source for identifying hidden source code dependencies. In: SOFSEM 2015: Theory and Practice of Computer Science: 41st International Conference on Current Trends in Theory and Practice of Computer Science, Pec pod Sněžkou, Czech Republic, January 24–29, 2015. Proceedings 41, Springer, pp 449–462 (2015)

[63] Lam, M.O., Hollingsworth, J.K.: Fine-grained floating-point precision analysis. Int. J. High Perform. Comput. Appl. **32**(2), 231–245 (2018)

[64] Lam, M.O., Hollingsworth, J.K., de Supinski, B.R., Legendre, M.P.: Automatically adapting programs for mixed-precision floating-point computation. In: Proceedings of the 27th international ACM conference on International conference on supercomputing, pp. 369–378 (2013)

[65] Lin, J., Liu, Y., Zeng, Q., Jiang, M., Cleland-Huang, J.: Traceability transformed: Generating more accurate links with pre-trained bert models. In: 2021 IEEE/ACM 43rd International Conference on Software Engineering (ICSE), IEEE, pp. 324–335 (2021)

[66] Linsbauer, L., Angerer, F., Grünbacher, P., Lettner, D., Prähofer, H., Lopez-Herrejon, R.E., Egyed, A.: Recovering feature-to-code mappings in mixed-variability software systems. In: 2014 IEEE International Conference on Software Maintenance and Evolution, pp. 426–430 (2014)

[67] Marchezan, L., Assunção, W., Herac, E., Keplinger, F., Egyed, A., Lauwerys, C.: Fulfilling industrial needs for consistency among engineering artifacts. In: 45th International Conference on Software Engineering (ICSE)-Software Engineering in Practice (SEIP), p. 12 (2023)

[68] Marques, A., Ramalho, F., Andrade, W.L.: Trl: a traceability representation language. In: Proceedings of the 30th Annual ACM Symposium on Applied Computing, pp. 1358–1363 (2015)

[69] Martins, L.E.G., Gorschek, T.: Requirements engineering for safety-critical systems: a systematic literature review. Inf. Softw. Technol. **75**, 71–89 (2016)

[70] Meskens, J., Luyten, K., Coninx, K.: Jelly: A multi-device design environment for managing consistency across devices. In: International Conference on Advanced Visual Interfaces, pp. 289–296. Association for Computing Machinery, New York, NY, USA (2010)

[71] Meyers, S.: Difficulties in integrating multiview development systems. IEEE Softw. **8**(1), 49–57 (1991)

[72] Missaoui, S., Mhenni, F., Choley, J.Y., Nguyen, N.: Verification and validation of the consistency between multi-domain system models. In: Annual IEEE International Systems Conference (SysCon), pp. 1–7 (2018)

[73] Noyer, A., Iyenghar, P., Pulvermueller, E., Pramme, F., Bikker, G.: Traceability and interfacing between requirements engineering and uml domains using the standardized reqif format. In: 2015 3rd International Conference on Model-Driven Engineering and Software Development (MODELSWARD), IEEE, pp. 1–6 (2015)

[74] Nurmuliani, N., Zowghi, D., Powell, S.: Analysis of requirements volatility during software development life cycle. In: Australian Software Engineering Conference, pp. 28–37 (2004)

[75] Nuseibeh, B., Kramer, J., Finkelstein, A.: Expressing the relationships between multiple views in requirements specification. In: Proceedings of 1993 15th International Conference on Software Engineering, pp. 187–196 (1993)

[76] Nuseibeh, B., Kramer, J., Finkelstein, A.: A framework for expressing the relationships between multiple views in requirements specification. IEEE Trans. Softw. Eng. **20**(10), 760–773 (1994)

[77] Pasqual, M.C., de Weck, O.L.: Multilayer network model for analysis and management of change propagation. Res. Eng. Des. **23**(4), 305–328 (2012)

[78] Pohl, K.: Requirements Engineering: Fundamentals, Principles, and Techniques. Springer Publishing Company, Incorporated (2010)

[79] Ponta, S.E., Plate, H., Sabetta, A.: Detection, assessment and mitigation of vulnerabilities in open source dependencies. Empir. Softw. Eng. **25**(5), 3175–3215 (2020)

[80] Qi, Q., Terkaj, W., Urgo, M., Jiang, X., Scott, P.: A mathematical foundation to support bidirectional mappings between digital models: an application of multi-scale modelling in manufacturing. Proc. R. Soc. A **478**(2264), 20220156 (2022)

[81] Qiu, X., Cen, W., Qian, Z., Peng, Y., Zhang, Y., Lin, X., Zhou, J.: Real-time constrained cycle detection in large dynamic graphs. Proc. VLDB Endow. **11**(12), 1876–1888 (2018)

[82] Quigley, M., Conley, K., Gerkey, B., Faust, J., Foote, T., Leibs, J., Wheeler, R., Ng, A.Y., et al.: Ros: an open-source robot operating system. In: ICRA workshop on open source software, Kobe, Japan, vol. 3, p. 5 (2009)

[83] Rabeau, S., Dépincé, P., Bennis, F.: Collaborative optimization of complex systems: a multidisciplinary approach. Int. J. Inter. Des. Manuf. **1**(4), 209–218 (2007)

[84] Raţiu, C.C., Assunção, W.K.G., Haas, R., Egyed, A.: Reactive links across multi-domain engineering models. In: 25th International Conference on Model Driven Engineering Languages and Systems, ACM, pp. 76–86 (2022)

[85] Rahimi, M., Goss, W., Cleland-Huang, J.: Evolving requirements-to-code trace links across versions of a software system. In: 2016 IEEE International Conference on Software Maintenance and Evolution (ICSME), pp. 99–109 (2016)

[86] Raţiu, C.C., Mayr-Dorn, C., Assunção, W.K., Egyed, A.: Taming cross-tool traceability in the wild. In: 2023 IEEE 31st International Requirements Engineering Conference (RE), IEEE, pp. 233–243 (2023)

[87] Reinert, J., Ritter, N.: Applying eca-rules in db-based design environments. In: CAD, pp. 188–201 (1998)

[88] Reiss, S.: Incremental maintenance of software artifacts. IEEE Trans. Softw. Eng. **32**(9), 682–697 (2006)

[89] Rubio-González, C., Nguyen, C., Nguyen, H.D., Demmel, J., Kahan, W., Sen, K., Bailey, D.H., Iancu, C., Hough, D.: Precimonious: Tuning assistant for floating-point precision. In: Proceedings of the international conference on high performance computing, networking, storage and analysis, pp. 1–12 (2013)

[90] Rubio-González, C., Nguyen, C., Mehne, B., Sen, K., Demmel, J., Kahan, W., Iancu, C., Lavrijsen, W., Bailey, D.H., Hough, D.: Floating-point precision tuning using blame analysis. In: Proceedings of the 38th International Conference on Software Engineering, pp. 1074–1085 (2016)

[91] Samer, R., Stettinger, M., Atas, M., Felfernig, A., Ruhe, G., Deshpande, G.: New approaches to the identification of dependencies between requirements. In: 2019 IEEE 31st International Conference on Tools with Artificial Intelligence (ICTAI), IEEE, pp. 1265–1270 (2019)

[92] Savić, M., Rakić, G., Budimac, Z., Ivanović, M.: A language-independent approach to the extraction of dependencies between source code entities. Inf. Softw. Technol. **56**(10), 1268–1288 (2014)

[93] Schwarz, H., Ebert, J., Winter, A.: Graph-based traceability: a comprehensive approach. Softw. Syst. Model. **9**, 473–492 (2010)

[94] Seibel, A., Neumann, S., Giese, H.: Dynamic hierarchical mega models: comprehensive traceability and its efficient maintenance. Softw. Syst. Model. **9**, 493–528 (2010)

[95] Seibel, A., Hebig, R., Giese, H.: Traceability in model-driven engineering: efficient and scalable traceability maintenance. Softw. Syst. Traceabil. **5**, 215–240 (2012)

[96] Sigelman, B.H., Barroso, L.A., Burrows, M., Stephenson, P., Plakal, M., Beaver, D., Jaspan, S., Shanbhag, C.: Dapper, a large-scale distributed systems tracing infrastructure. Tech. rep., Google, Inc., https://research.google.com/archive/papers/dapper-2010-1.pdf (2010)

[97] Song, B., Zurita, N.S., Zhang, G., Stump, G., Balon, C., Miller, S.W., Yukish, M., Cagan, J., McComb, C.: Toward hybrid teams: A platform to understand human-computer collaboration during the design of complex engineered systems. Design Society Conference, Cambridge University Press **1**, 1551–1560 (2020)

[98] Sreedhar, V.C., Gao, G.R., Lee, Y.F.: Identifying loops using dj graphs. ACM Trans. Programm. Lang. Syst. **18**(6), 649–658 (1996)

[99] Strasunskas, D., Hakkarainen, S.E.: Domain model-driven software engineering: a method for discovery of dependency links. Inf. Softw. Technol. **54**(11), 1239–1249 (2012)

[100] Sun, C., Xia, S., Sun, D., Chen, D., Shen, H., Cai, W.: Transparent adaptation of single-user applications for multi-user real-time collaboration. ACM Trans. Comput.-Hum. Inter. **13**(4), 531–582 (2006)

[101] Tang, A., van Vliet, H.: Modeling constraints improves software architecture design reasoning. In: Joint Working IEEE/IFIP Conference on Software Architecture European Conference on Software Architecture, pp. 253–256 (2009)

[102] Tang, D.B., Yin, L.L., Wang, Q., Ullah, I., Zhu, H.H., Leng, S.: Workload-based change propagation analysis in engineering design. Concurr. Eng. **24**(1), 17–34 (2016)

[103] Tarawaneh, R.M., Keller, P., Ebert, A.: A general introduction to graph visualization techniques. In: Visualization of Large and Unstructured Data Sets: Applications in Geospatial Planning, Modeling and Engineering-Proceedings of IRTG 1131 Workshop 2011, Schloss Dagstuhl-Leibniz-Zentrum fuer Informatik (2012)

[104] Tarjan, R.: Depth-first search and linear graph algorithms. SIAM J. Comput. **1**(2), 146–160 (1972)

[105] Tarjan, R.: Testing flow graph reducibility. In: Proceedings of the fifth annual ACM symposium on Theory of computing, pp. 96–107 (1973)

[106] Tominski, C., Abello, J., Schumann, H.: Cgv-an interactive graph visualization system. In: Computers & Graphics 33.6, pp. 660–678 (2009)

[107] Tröls, M.A., Mashkoor, A., Egyed, A.: Collaboratively enhanced consistency checking in a cloud-based engineering environment. In: ACM SIGCHI Symposium on Engineering Interactive Computing Systems, ACM (2019)

[108] Tröls, M.A., Mashkoor, A., Egyed, A.: Instant distribution of consistency-relevant change information in a hierarchical multi-developer engineering environment. In: 36th Annual ACM Symposium on Applied Computing, ACM, pp. 1572–1575, (2021) 10.1145/3412841.3442127

[109] Ullah, I., Tang, D., Wang, Q., Yin, L.: Exploring effective change propagation in a product family design. J. Mech. Des. **139**(12), 121101 (2017)

[110] Vidal, M., Massoni, T., Ramalho, F.: A domain-specific language for verifying software requirement constraints. Sci. Comput. Program. **197**, 102509 (2020)

[111] Wang, W., Dumont, F., Niu, N., Horton, G.: Detecting software security vulnerabilities via requirements dependency analysis. IEEE Trans. Softw. Eng. **48**(5), 1665–1675 (2020)

[112] Warmer, J.B., Kleppe, A.G.: The Object Constraint Language: Getting Uour Models Ready for MDA. Addison-Wesley Professional, London (2003)

[113] White, R., Krinke, J., Tan, R.: Establishing multilevel test-to-code traceability links. In: Proceedings of the ACM/IEEE 42nd International Conference on Software Engineering, pp. 861–872 (2020)

[114] Yamazaki, I., Tomov, S., Dong, T., Dongarra, J.: Mixed-precision orthogonalization scheme and adaptive step size for improving the stability and performance of ca-gmres on gpus. In: International Conference on High Performance Computing for Computational Science, Springer, pp. 17–30 (2014)

